# Sequence-to-function deep learning frameworks for engineered riboregulators

**DOI:** 10.1038/s41467-020-18676-2

**Published:** 2020-10-07

**Authors:** Jacqueline A. Valeri, Katherine M. Collins, Pradeep Ramesh, Miguel A. Alcantar, Bianca A. Lepe, Timothy K. Lu, Diogo M. Camacho

**Affiliations:** 1 Wyss Institute for Biologically Inspired Engineering, Harvard University, Boston, MA 02115 USA; 2 Institute for Medical Engineering and Science and Department of Biological Engineering, Massachusetts Institute of Technology, Cambridge, MA 02139 USA; 3 Department of Brain and Cognitive Sciences, Massachusetts Institute of Technology, Cambridge, MA 02139 USA; 4 Synthetic Biology Group, Research Laboratory of Electronics, Massachusetts Institute of Technology, Cambridge, MA 02139 USA

**Keywords:** Machine learning, Synthetic biology

## Abstract

While synthetic biology has revolutionized our approaches to medicine, agriculture, and energy, the design of completely novel biological circuit components beyond naturally-derived templates remains challenging due to poorly understood design rules. Toehold switches, which are programmable nucleic acid sensors, face an analogous design bottleneck; our limited understanding of how sequence impacts functionality often necessitates expensive, time-consuming screens to identify effective switches. Here, we introduce Sequence-based Toehold Optimization and Redesign Model (STORM) and Nucleic-Acid Speech (NuSpeak), two orthogonal and synergistic deep learning architectures to characterize and optimize toeholds. Applying techniques from computer vision and natural language processing, we ‘un-box’ our models using convolutional filters, attention maps, and in silico mutagenesis. Through transfer-learning, we redesign sub-optimal toehold sensors, even with sparse training data, experimentally validating their improved performance. This work provides sequence-to-function deep learning frameworks for toehold selection and design, augmenting our ability to construct potent biological circuit components and precision diagnostics.

## Abstract (web summary)

The design of synthetic biology circuits remains challenging due to poorly understood design rules. Here the authors introduce STORM and NuSpeak, two deep-learning architectures to characterize and optimize toehold switches.

## Introduction

Advances in synthetic biology have shifted paradigms in biotechnology by drawing inspiration from nature. While researchers have successfully isolated and adapted templates from naturally occurring circuit parts—such as inducible promoters, terminators, and riboswitches—forward-engineering of components remains challenging^1^. The workflow to develop a single biological circuit may require weeks of screening and fine-tuning in order to perform a desired function. As such, there is a strong need for in silico screening of circuit parts in order to ease integration of both naturally occurring and redesigned synthetic components into engineered biological systems.

In order to address the complexity of prediction and design of biological circuit parts, computational tools can aid in modeling and redesigning nucleic acid sensors, such as riboswitches. Since the discovery of naturally occurring riboregulators—RNA molecules that alter their translation rate in the presence of small molecules or nucleic acids via changes in secondary structure^2,3^—synthetic biologists have co-opted these circuit components for a variety of uses, from synthetic gene circuit construction^4,5^ to gene regulation^6,7^.

**Fig. 1:**
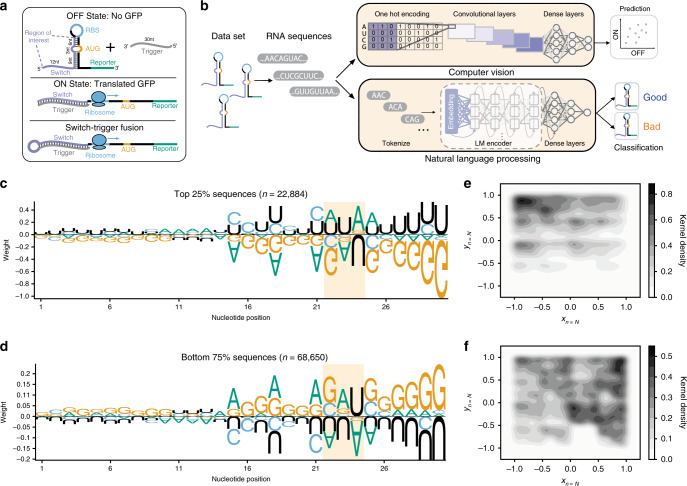
Deep learning frameworks are needed to accurately stratify toehold switches. **a** Toehold switches modify their secondary structure in response to the presence of a complementary RNA molecule known as a trigger. In absence of the trigger, the Shine-Dalgarno sequence, or ribosome binding site (RBS), remains inaccessible and the reporter protein is not translated (OFF state). Upon binding of the trigger to the switch, the hairpin melts, allowing ribosome recruitment to the Shine–Dalgarno sequence and subsequent translation of the downstream reporter GFP protein (ON state). A modified ON state switch was built for experimental testing so that one molecule could be tested with trigger and switch fused together. **b** Two deep learning frameworks employing different strategies from computer vision and natural language processing were used to classify and predict toehold switch performance. **c** Sequence logos were calculated for the top 25% (*N* = 22,884) and **d** bottom 75% (*N* = 68,650) of sequences according to the experimental ON/OFF ratios. Weight corresponds to the log_2_ of each nucleotide probability normalized by the background frequency of that nucleotide in the set of all experimentally tested toeholds. Highlight indicates the motif found in positions 22–24 (Supplementary Fig. S1). **e** Chaos-game-representations (CGR) for the top 25% (*N* = 22,884) and **f** bottom 75% (*N* = 68,650) of toehold switches were calculated to visualize macroscopic sequence patterns, where darker regions correspond to an enrichment of sequences in that CGR locus. The sequence logos, CGR plots, and biophysical properties (Supplementary Fig. S3), however, were insufficient to characterize toehold performance on their own.

The toehold switch, a particularly versatile synthetic riboregulator, is able to detect and respond to the presence of RNA molecules via linear–linear hybridization interactions^8^. The anatomy of a typical toehold switch consists of an unstructured RNA strand, followed by a hairpin that sequesters the Shine–Dalgarno sequence such that a downstream protein coding sequence is not translated when the switch is in the OFF state (Fig. 1a, Supplementary Table S1). The toehold can be flexibly programmed such that the unstructured switch RNA and ascending stem of the hairpin (positions 1–30) are complementary to an arbitrary trigger 30-nucleotide RNA sequence—which upon hybridization to the switch subsequently melts the hairpin (toehold ON state), thereby exposing the Shine–Dalgarno sequence to the ribosome, which then initiates translation of the sequestered coding sequence. This fundamentally inducible nature of toehold switches has led to their successful use in both low-cost, freeze-dried, paper-based diagnostics^9–11^, as well as multiplexable components in complex genetic circuits with low crosstalk^12,13^.

Although toehold switches have become an effective and modular component of the synthetic biology toolkit, broad understanding of switch design has been limited by the small number of available toehold switches and the lack of effective design rules for achieving optimal performance. Sequence-based computational tools, which take into account thermodynamic equilibria and hybridization energies, have been developed to predict RNA secondary structure prior to experimental validation^14–17^. However, when applied to multi-state toehold switches, these tools can lack predictive power and require time-intensive experimental screening, with correlations as low as 0.22 between predicted and measured efficacy^17^. Accordingly, an improved and interpretable prediction framework would rapidly expand the applicability of these versatile riboregulators for biological applications.

To improve toehold switch design and prediction, we took inspiration from the broader field of machine learning. Machine learning approaches have been applied successfully to systems and synthetic biology^1,18^ and in motif finding and DNA sequence prediction tasks^19–21^, among many other applications in biology and medicine. Today, most commonly used deep learning algorithms for synthetic biology are broadly derived from either computer vision or natural language processing (NLP) based approaches^22^. Convolutional neural nets (CNNs)^23^ comprise the backbone of most computer vision-based algorithms and excel at elucidating important sequence motifs in biological sequences, as convolutional layers offer a high level of interpretability. When CNNs are applied to images, consecutive convolutional layers learn increasingly abstract features; for example, in facial recognition tasks, edges and curves are learned in the first convolutional layer, followed by eyes, noses, and mouths, accumulating higher-order features through the layers^23^. As secondary structure is an important feature of toehold switches, a convolutional architecture may be able to function as a motif detector and learn additive features of motifs or partial motifs of the linear RNA sequence.

To learn nonlocal interactions and overcome potential limitations of CNNs applied to biologically relevant sequences, language models can be built using recurrent neural nets (RNNs)^24^, which can consider the entire sequence as a whole instead of fixed window lengths as in CNNs^25^. These RNNs can learn long-range dependencies amongst sequences and sequence motifs in an unsupervised manner by employing a serially connected chain of long short-term memory neurons (LSTMs)^26^, which learn a contextual representation of words in a sequence. RNNs, like other deep learning models, can be paired with natural language processing techniques such as tokenization and word embeddings. As a result, a composite RNN-language model (LM), herein referred to as the language model, can learn the broader “grammar” dictating the ordering of these words. Since language models have achieved state-of-the-art performance on human language tasks^27^, similar architectures may extend to biological language families such as RNA and DNA. Analogous to Latin being the parent language for all Indo-European romance languages, RNA architectures such as toeholds, riboswitches, and CRISPR gRNAs, may be able to be thought of as distinct descendant-languages that lie within the parent RNA language family.

In this work, we construct two complementary yet orthogonal deep learning models to uncover design rules in toeholds given that both frameworks have unique advantages for sequence modeling and machine learning-assisted sequence design. In addition, the two trained models lend themselves to a “consensus” approach, where we can ensure any biological meaning derived from our models are not artifacts of a particular architecture. Influenced by work indicating that hybrid CNN/RNN architectures can boost model accuracy for biological problems given sufficient training data^28^, we develop an integrated pipeline with both models to exploit the “wisdom of crowds”^29^. Given the large amounts of training data required by common deep learning approaches, we partner with Angenent-Mari et al.^30^ to design and generate a dataset of toeholds that are complementary to human genomic elements, RNA viruses, and random sequences. The toeholds are experimentally characterized with a coupled flow-cytometry and deep-sequencing pipeline, inspired by previous flow-seq methodologies^31^. While Angenent-Mari et al.^30^ evaluate a variety of deep learning architectures trained on thermodynamic parameters, raw nucleic acid sequences, and base-pair complementarity matrices, we extend this investigation to explain, adapt, and tune the predictions made by two sequence-to-function models, a traditional CNN-based regressor and a language model-based classifier (Fig. 1b). Using previously established explainable AI techniques, we examine motifs or partial motifs detected by the convolutional filters as well as positional importance of nucleotides. We then provide examples of utilizing our deep neural networks synergistically to characterize toeholds that sense pathogenic genomes. Finally, we extend both models to rationally re-engineer poorly-performing toeholds, creating the NLP-based, nucleotide-centric language model Nucleic Acid Speech (NuSpeak) and a CNN-based Sequence-based Toehold Optimization and Redesign Model (STORM). These models achieve divergent purposes, as they optimize both sensors for pathogens and toeholds as synthetic circuit components, respectively. Sequence-to-function frameworks such as the ones proposed here enable researchers to rapidly cycle through many possible design choices and select circuitries with optimal performance, while elucidating underlying design principles for riboregulators. Application of these frameworks to other training data and training tasks is both direct and desirable, as methods such as transfer learning allow for translation between different experimental set-ups. Intersecting the cutting-edge features and techniques of both deep learning and synthetic biology holds important implications for human health and biotechnology.

## Results

### Nucleotide over-representation in good and poor toeholds

A dataset of 244,000 toehold switches, including sequences tiled from viruses and the human genome as well as random sequences (see “Methods”), was designed jointly with Angenent-Mari et al.^30^, with 91,534 switches meeting well-defined quality control criteria after experimental characterization. We conducted a traditional bioinformatics over-representation investigation and started by splitting sequences into the top 25th and bottom 75th percentile based on the experimental ON/OFF values. We then visualized each average position weight matrix as sequence logos, normalized with respect to the background nucleotide probability distribution (Fig. 1c, d). Stratification into high- and low-performing sequences shows differential nucleotide composition immediately before the SD sequence, with uracil appearing over-represented and guanine under-represented in good toeholds (top 25%) and the converse true for poorly-performing toeholds (bottom 75%). An enrichment for NUA in positions 22–24 is highlighted in the top-performing logo with 10.3% of the top 25% of sequences containing the NUA motif. This triplet corresponds to the three-nucleotide bulge directly opposite from the AUG start codon, suggesting that high-performing sequences may have an NUA at this bulge to prevent hybridization to the start codon (Supplementary Fig. S1). Interestingly, 38.6% of top sequences contain a U in position 22 and 35.3% of top sequences contain an A in position 23, implying that single nucleotides belonging to the NUA motif are more prevalent than the complete motif. In addition, positions 15, 18, and 21 show over- and under-representation of adenine in bad and good toeholds respectively. These positions are directly opposite in the toehold stem to the first nucleotides of the only three in-frame coding triplets of the descending stem. This suggests that U in positions 51, 54, and 57 is detrimental to toehold performance, possibly due to the fact that the N-terminus of the reporter protein cannot tolerate an in-frame stop codon (i.e., UAA, UGA), which would terminate translation of the reporter protein.

To further understand how changes to the coding part of the sequence (positions 51–59) affect toehold performance, we conducted a broader analysis of the in-frame amino acids (Supplementary Fig. S2) and as expected found that in-frame stop codons occurred less often at the N-terminus of high-performing sequences. In addition, though an unstructured linker region separates the switch from the reporter gene, toehold sequences appear sensitive to changes to in-frame amino acids at the N-terminus of the protein. Small hydrophobic amino acids such as valine, alanine, and glycine appear more often in the N-terminus of high-performing sequences than low-performing ones; top sequences also appear to contain less proline at the N-terminus, suggesting a slight preference for amino acids lacking a secondary amine. However, due to the nature of the study, it is difficult to disentangle whether the observed enrichments for certain amino acids are due to structural changes at the RNA level or protein level, or perhaps due to differences in tRNA abundance.

To elucidate any macroscopic sequence patterns between good and bad toeholds, we utilized chaos-game-representations (CGR) which provide an informative and lossless encoding scheme by which any nucleic-acid sequence can be fully represented with just three numbers (length, *X*_*CGR*_, and *Y*_*CGR*_) (Fig. 1e, f). Since all toeholds within our dataset have the same length of 59 nucleotides, a two-dimensional CGR vector, consisting only of *X*_*CGR*_, and *Y*_*CGR*_, is sufficient to represent any given toehold sequence. Furthermore, since the trigger-binding region (first 30 nucleotides) is the principal variable region across all the toeholds, we computed its two-dimensional CGR coordinates, and observed an enrichment of A-rich codons amongst good performers (Fig. 1e).

### Biophysical properties are not predictive for top switches

The large size of the dataset allowed us to conduct an unbiased evaluation of toeholds’ biophysical properties suggested by other studies^14,17^. As previous reports have indicated that GC content is important for the strength of the ON and OFF state stabilities^8^, we compared the GC content distributions for top-performing sequences to that of all sequences (Supplementary Fig. S3A). Our results suggest that successful toeholds may have a range of acceptable GC content between 20 and 60%, implying a necessity for some A–U base pairing in the switch.

In addition, multiple secondary structure prediction tools rely on thermodynamic modeling^14–17^; for example, the NUPACK software package calculates the equilibrium Gibbs free energy values for many possible secondary structures based on a provided RNA sequence, and reports to the user the most likely structure based on a minimum free energy (MFE) determination^14^. Because MFE is thought to be a predictive metric^8,14,16,17^, we assessed the MFE distribution in top-performing sequences against that of all sequences (Supplementary Fig. S3B). High-performing sequences had a statistically significantly higher MFE distribution than the set of all sequences, possibly due to an over-stable hairpin that does not readily melt in the presence of the trigger. Although top sequences exhibit statistically significant shifts in both GC content and MFE distributions, these properties lack sufficient predictive power due to their broad range of acceptable values.

### Interpretability of deep learning framework predictions

As no single or combinations of biophysical properties were found to be sufficiently predictive of switch performance, deep learning sequence-to-function models using both CNNs and RNNs were built to predict toehold behavior. Given the recent advancements in both accuracy and accessibility of deep learning^1,32^, a CNN^23^ was constructed to take RNA sequences as inputs, employing two convolutional layers to identify plausible motifs and partial motifs in the input sequences^33^ (Fig. 1b). Following the convolutional layers, the model employs a multi-layer perceptron (MLP) with three dense layers, where every node in a given layer is connected to every node in the previous layer, to synthesize the features from the convolutions to output an ON and OFF prediction for each toehold sequence (see “Methods”).

**Fig. 2:**
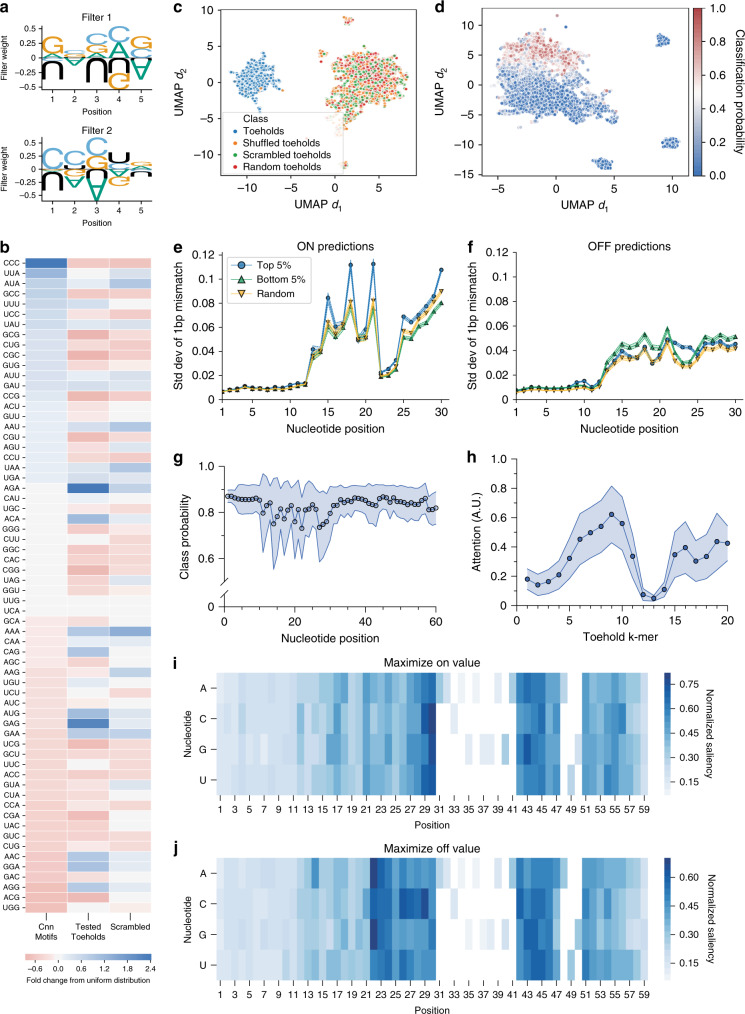
Interpretable machine learning tools can extract intrinsic characteristics of toehold sequences. **a** To understand the sequence patterns registered by the convolutional neural network (CNN)-based model, the learned filter weights for each of the 10 filters of width 5 in the first convolutional layer can be visualized as sequence logos similar to position weight matrices, with two examples shown here. **b** To understand trends in these filters, the frequency of all three-mers in filters from 20 separately trained models were examined relative to their expected value in a uniformly distributed collection of three-mers, alongside bootstrapped sets of the experimental toeholds and a collection of scrambled toeholds. **c** The language model (LM) learns an embedding of synthetic toehold sequences (*N* = 5000) distinct from other nucleic acid sequences, including scrambled toeholds. **d** The LM embedding of 5000 experimentally tested toehold sequences cluster according to predicted classes. **e** To investigate the relative importance of each position in the toehold sequence, the effect of single base pair in silico mutations were evaluated on the CNN-based model for both ON and (**f**) OFF predictions, as well as for (**g**) language model predictions. For the CNN-based predictions, shaded area indicates the mean ± 95% confidence interval of 2500 sequences for the top 5% of sequences (blue circle), bottom 5% of sequences (green triangle), and a random sample of sequences (yellow triangle). For LM-based predictions, shaded area indicates the mean ± standard deviation of 5000 sequences. **h** White box tools were used to evaluate the “attention” of the language model for 5000 sequences randomly selected from the held-out test set, where attention represents the normalized contribution of each k-mer in a sequence for the class that the sequence belongs to. Shaded area indicates mean ± standard deviation. **i** In parallel, saliency maps were generated for 100 sequences to elucidate the importance of each nucleotide to the CNN-based model’s ON and **j** OFF predictions, with saliency serving as a proxy for the model’s attention to the nucleotide at that position.

Given the vast number of weights and nonlinear functions that form the backbone of neural networks, it can be challenging to deduce why a model made the predictions it did^32^. While recent work such as soft explainable decision trees^34^ have enabled researchers to look inside this “black-box” by using a neural network to train a decision tree, we chose to visualize weights and activations of our trained model directly^35^. Taking inspiration from work in the fields of image recognition and genomics^23,36–38^, we “un-boxed” the first convolutional layer to visualize the features our model deemed important by interpreting the filter weights learned from input sequences as sequence logos (Fig. 2a). To understand trends in convolutional filters, we trained the CNN 20 times and explored the frequencies of three-mers in the resulting ensemble of filters (Fig. 2b). When compared to the expected value under a uniform distribution, the “CCC” three-mer occurs almost 2.5× more often than expected, suggesting the model may learn this motif for improved prediction. Additionally, the trained model learns to ignore sequences over-represented in the experimental toeholds—AGA and GAG, for example—that are part of the SD sequence and thereby conserved across all toeholds, indicating that the CNN learns to discern which positions are significant during the training process.

Similarly, we also constructed an encoder-decoder architecture to learn the language of toehold sequences, where each k-mer is treated as a “word” or “token” and each toehold sequence is a “sentence”. The LM encoder takes an RNA sequence that has been tokenized, or split into k-mers, as its input, and passes the tokens through an embedding layer, which maps each k-mer to a vector representation. The vector is then passed through a four-layer quasi-recurrent neural network (QRNN)^39^ to return a 400-dimensional vector. In this LM pre-training step^40^, the encoder learns a meaningful, context-dependent representation for each unique token found within the input corpus, which can then be extended with a linear classification layer to predict if a given toehold is good or bad. When augmented with a decoder that maps vectors back into tokens, the complete LM can generate meaningful sequences of arbitrary length within the language space.

In order to generate a sufficiently diverse and large toehold corpus, we first trained a LM on a set of 4 million synthetic toeholds generated in silico (see “Methods”). To determine whether the LM had learned a meaningful representation of toeholds, we mapped the 400-dimensional representation of a toehold sequence onto a reduced dimension manifold with UMAP^41^ (Fig. 2c), and compared to both scrambled and shuffled—which are scrambled after tokenization—input sequences, as well as random sequences. We observed no overlap between real toehold sequences and controls on the two-dimensional manifold, suggesting that the LM captured the importance of motif order in a toehold sequence.

Given the success of the LM classifier on synthetic sequences, we used the toehold dataset from Angenent-Mari et al.^30^ to train a sequence classifier. The UMAP manifold of 5000 randomly sampled sequences color-coded by the predicted classes suggests that the classifier has learned to bifurcate good and bad performers (Fig. 2d). Unsurprisingly, sequences with classification probabilities close to 0.5 populate the decision boundary. This classifier is ~3.7 fold and ~6.2 fold more predictive than classifiers that use shuffled and scrambled toeholds, respectively, again reinforcing the notion that sequence motif order is important for differentiating toehold performance and demonstrating that the model has learned more than k-mer frequencies.

### Positional importance of nucleotides and model attention

To understand how variations in a toehold sequence might affect model predictions, we conducted a mutagenesis scan across sets of 2500 random experimental toeholds. For each position in the toehold, we mutated all four possible base pairs at each position and calculated the standard deviation of the CNN-based model’s ON (Fig. 2e) and OFF (Fig. 2f) predictions. Spikes in effect sizes at positions 15, 18, and 21, mirror the important positions in the sequence logos (Fig. 1c, d), suggesting the model learned positional importance of nucleotides. In parallel, a set of 500 randomly chosen good toeholds were serially mutated at each position with a random nucleotide and fed back into the LM to compute classification probabilities (Fig. 2g). Echoing the prior mutational analyses, positions 26–30 were shown to have the biggest impact on toehold performance.

To ascertain the models’ decision-making processes and further identify important regions in the toehold sequence, we first calculated the language model’s intrinsic self-attention on a set of 5000 randomly sampled toeholds (Fig. 2h). The self-attention map suggests that the ascending stem, specifically the last 12 nucleotides of the switch region, has the biggest influence on classification decision. Unsurprisingly, given that the RBS and start codon do not vary across the dataset, the model learns to ignore these regions. These results are mirrored in the saliency maps computed on the CNN-based model, where we evaluated the importance of each position in 100 random sequences towards maximizing the ON value (Fig. 2i) and minimizing the OFF value (Fig. 2j). Here, a higher saliency, computed by summing gradients across nucleotides at each position (see “Methods”), indicates that the nucleotide was considered to be more influential in the model’s ON or OFF prediction process. To understand if the sequence saliency varies with the experimental values of the ON or OFF prediction, saliency maps for sets of high- and poorly-performing toeholds were evaluated (Supplementary Fig. S4). Poorly-performing toehold maps show similarly low activation in the first 12 nucleotides as their high-performing counterparts, suggesting that the model learns the relationship between different regions of toeholds and predicted function.

### Models predict toehold performance even with sparse data

**Fig. 3:**
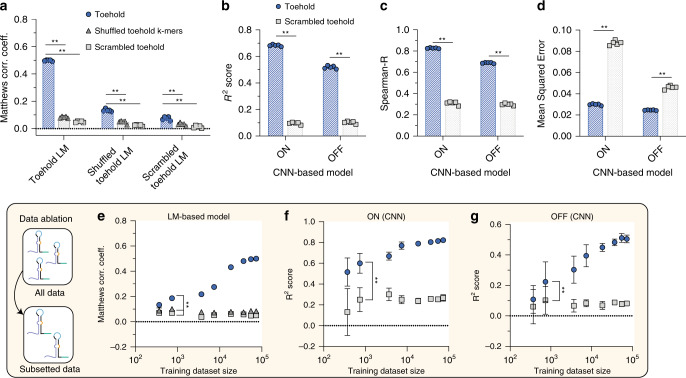
Models can accurately predict toehold performance with sparse data. **a** The language model (LM) trained on toehold embeddings only accurately classifies real toeholds (blue circle), not shuffled (gray triangle, *p* = 2.45 × 10^−14^) or scrambled sequences (gray square, *p* = 5.03 × 10^−15^), as assessed by Matthews Correlation Coefficient (MCC). While language models trained on shuffled toeholds and scrambled toeholds are also more accurate for real than shuffled (*p* = 1.18 × 10^−6^, *p* = 4.41 × 10^−4^) and more accurate for real than scrambled toeholds (*p* = 1.58 × 10^−8^, *p* = 2.01 × 10^−5^), they fail to achieve the accuracy of the LM trained on real toeholds. **b** The convolutional neural network (CNN)-based model predictions for both ON and OFF are significantly higher than predictions on scrambled toeholds in five-fold cross validation, evaluated with R^2^ for ON and OFF (*p* = 2.97 × 10^−14^, *p* = 4.19 × 10^−12^), **c** Spearman correlation (*p* = 1.46 × 10^−12^, *p* = 9.09 × 10^−13^), and (**d**) MSE (*p* = 9.59 × 10^−12^, *p* = 2.12 × 10^−9^). For (**a**–**d**), *N* = 5 cross validation folds. **e** Data ablation studies were performed with both the LM and CNN-based model, evaluating both real toeholds (blue circle), shuffled sequences (gray triangle) and scrambled sequences (gray square). LM performance does not drop off steeply with half as much data and continues to perform better on real vs. shuffled (*p* = 9.23 × 10^−9^) and real vs. scrambled (*p* = 6.65 × 10^−10^) with just 736 training examples. For (**e**), *N* = 5 trials. **f** Similarly, the CNN-based model performance does not drop off steeply for either ON or (**g**) OFF prediction with half as much data and has significantly different *R*^2^ values at a training size of 736 samples for both ON and OFF predictions (*p* = 7.23 × 10^−7^, *p* = 0.028). For (**f**, **g**), *N* = 10 trials. For all panels, error bars represent mean ± standard deviation and all tests are two-tailed t-tests.

Expanding the comparison between both model architectures on the same task, we systematically evaluated how the language model classifies good and bad toeholds for three ON/OFF thresholds (Fig. 3a, Supplementary Fig. S5), in addition to how the CNN-based model predicts the ON and OFF states as continuous values (Fig. 3b–d). Interestingly, we observed that all models had higher correlative metrics based on switch ON values alone. These results suggest that the models are able to learn features distinguishing a high ON value more readily, possibly resulting from variance in the OFF state due to autofluorescence not being subtracted out^8^. Since the ON/OFF ratio is an internally normalized performance metric and the fluorescence data were sorted into four bins, we chose an ON/OFF ratio of top 25% as the optimal threshold combination for the language model. As an additional validation experiment, toeholds corresponding to those obtained by tiling viral genomes were held-out during the classifier training phase (Supplementary Fig. S6). We subsequently fed these sequences into the trained model and scored the predictions, and observed similar performance (average MCC~0.50) across toeholds sensing 20 different viral genomes.

We also evaluated our models against more established, off-the-shelf methods (see “Methods”). When comparing the LM with other commonly used NLP architectures based on either term-frequency inverse document frequency (tf-idf)^42^ or skip-gram based word-embeddings^43^ (Supplementary Fig. S7), we observed that skip-gram-based word-embedding models were on-average ~1.8 fold more predictive than the tf-idf models. The LM significantly outperformed all other word-embedding-based architectures, including a bidirectional LSTM^44^ paired with a self-attention layer^45^, considered state-of-the-art for NLP sentiment classification tasks.

To elucidate whether the models were saturated, we computed learning curves for both sets of architectures (Fig. 3e–g). Despite training on small dataset sizes (*N* = 736), both models were able to generate meaningful predictions (MCC~0.19 and *R*^2^~0.6) relative to scrambled and shuffled controls. We hypothesized that the low variance associated with the language model predictions is a direct consequence of pre-training on the in silico set of 4 million toehold sequences, which results in a stable word-embedding, while the CNN is likely robust to smaller datasets due to its convolutional architecture^46^. Other commonly used word-embedding architectures had significantly higher variance in predictive performance across all training sample sizes and do not appear to saturate (Supplementary Fig. S8). Collectively, these data demonstrate the power of these architectures to train on considerably less data than anticipated.

### Transfer learning and extending our models to unseen genomes

**Fig. 4:**
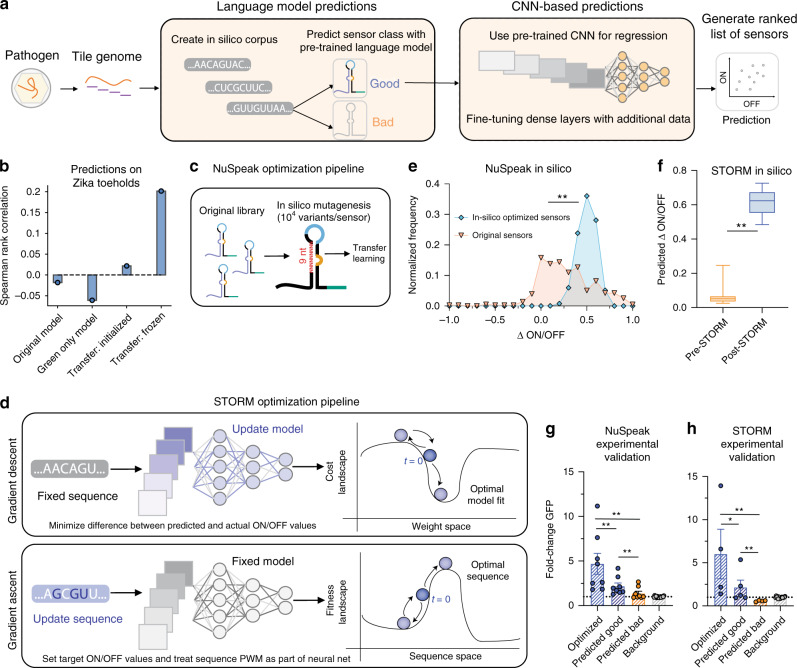
Prediction and optimization of toeholds can be used for diagnostic sensor development. **a** Diagnostic sensor optimization pipeline involves tiling the genome of a pathogen, creation of an in silico corpus, and utilizing both the natural language processing (NLP)-based and convolutional neural network (CNN)-based models to generate a list of sensors for experimental validation. **b** To balance the utility of the large Angenent-Mari et al.^30^ dataset with the nature of the Green et al. dataset^8^, which tested toeholds with free RNA as opposed to switch-trigger fusions, we used transfer learning to fine-tune the existing model and achieve a higher degree of correlation with the predictions on an external validation set of free-trigger Zika toeholds^10^ (*N* = 24). Data are shown for the CNN-based model rank predictions. **c** The NuSpeak optimization pipeline was designed to maintain base-pairing complementarity in the switch while producing all possible variants of positions 21–30. Sequences are then run through the consensus pipeline with fine-tuned models as above to produce a ranked list of sensors for a given region. **d** In contrast to traditional model training, which uses gradient descent to optimize the model’s weights from randomly initialized values (dark blue, time = 0) and predict the ON and OFF values for any given sequence, the CNN-based model can be inverted for gradient ascent of the sequence. Target ON and OFF values can be set, and the sequence space can be explored, starting at a given initialized sequence (dark blue, time = 0), to achieve those target values while the trained model remains fixed. Both optimization pipelines effectively improve the in silico ON/OFF ratio. **e** NuSpeak was used to optimize sequences with an increase in in silico ON/OFF ratio (blue circle) from the original ON/OFF ratio (orange triangle) for experimentally tested sequences (*N* = 354, *p* = 4.54 × 10^−33^). **f** Likewise, the Sequence-based Toehold Optimization and Redesign Model (STORM) was used to optimize experimental sequences, with center line indicating median and box limits indicating 25th and 75th quartile (*N* = 20, *p* = 3.33 × 10^−8^). For (**e**, **f**), a two-tailed Mann-Whitney *U* test was used. **g**, **h** Both pipelines can be used to predict and optimize SARS-CoV-2 viral RNA sensors, with experimentally validated toeholds performing as predicted. There is a significant increase in NuSpeak predicted bad (*N* = 8) and predicted good (*N* = 8) values (*p* = 0.026), as well as predicted good and optimized (*N* = 8) values (*p* = 0.020) and predicted bad and optimized values (*p* = 0.004). Likewise, there is a significant increase in STORM predicted bad (*N* = 4) and predicted good (*N* = 5) values (*p* = 0.010), as well as predicted good and optimized (*N* = 4) values (*p* = 0.089) and predicted bad and optimized values (*p* = 0.015). For (**g**, **h**), error bars indicate mean ± S.E.M and a one-tailed Mann–Whitney *U* test was used. Background was calculated by measuring the initial ON/OFF ratio. Source data are provided as a Source Data File.

Considering the unique advantages of both the language model and CNN architectures, we incorporated both architectures into a pipeline for designing toeholds that optimally detect any arbitrary nucleic-acid sequence, such as a fragment of a viral genome (Fig. 4a). While our models achieved robust performance on predicting toehold sensors tested by Angenent-Mari et al.^30^, we sought to ensure their generalizability for scoring toehold sensors that detect free trigger RNA, given that the utility of toehold diagnostics applications stem from their ability to bind and detect exogenous RNAs. We thus explored our model performance on a smaller set of 168 sequences from Green et al.^8^ that had been tested in a context containing free trigger RNA rather than with a fused trigger.

As the pre-trained LM achieved a Matthew’s Correlation Coefficient (MCC) of only 0.42 on the Green et al. dataset^8^, compared with 0.51 on the Angenent-Mari et al. dataset^30^, we hypothesized that a more predictive model could be constructed by fine-tuning our pretrained language model on toeholds tested in a free trigger context^8^, using transfer learning techniques to bridge the gap. Transfer learning allows for recycling of information, typically from a more general task to a more context-specific task which may have less training data available^47^. We incorporated the 168 free-trigger sequences from Green et al.^8^ as our second, smaller training set. As research has shown that transferring weights from any number of layers can improve the accuracy of a re-trained model^48^, we froze the first three QRNN layers of our pre-trained model, permitting only the weights in the fourth QRNN layer and classification layer to vary during training. This method achieved an MCC of 0.56 with a small held-out validation set, constituting a 33% improvement. As the CNN-based model showed a low *R*^2^ of 1.87e-4 on ON/OFF values predicted for the Green et al. sequences^8^, we then carried out the same transfer learning protocol for the CNN-based model by freezing the weights of the convolutional layers and allowing only the dense layer weights to vary during training. We observed an average *R*^2^ of 0.18 and an average Spearman correlation of 0.36 over five-fold cross validation, again with small held-out validation sets.

We were also interested in the performance of the models on an external validation set. We evaluated the rank correlation of the CNN-based model predictions on 24 unseen Zika toeholds from Pardee et al.^10^ (Fig. 4b). When compared to our previous model, a model trained only on the 168 free-trigger sequences, and a transfer learning model initialized but not frozen with the weights of the pre-trained model, we confirmed that freezing the weights achieves the highest rank correlation of around 0.2 compared to the negligible correlations achieved by other models. Though these correlations may seem low, the experimental setup for the Pardee et al. sequences^10^ was vastly different: the researchers use lacZ activity as a readout and add an amplification step before detection so true trigger concentrations are unknown. Similarly, the language model accurately classifies all 24 toeholds as being good, consistent with the in-depth in silico screening process undertaken by Pardee et al.^10^ to narrow a field of thousands of potential sequences to the 24 toeholds, based on rigorous secondary structure analyses, BLAST searches, and multiple constraints on the linear sequence. Unlike a model trained only on Green et al. sequences^8^, which is likely underfit given the sparse training data, we can achieve meaningful biological predictions by conducting this fine-tuning step. Armed with these more predictive models, we integrated both the re-trained language model and re-trained CNN-based model into a pipeline that tiles any genomic sequence and returns a list of all possible toehold sensors ranked by their predicted ON/OFF value (Fig. 4a).

To illustrate the value of our approach, and in a proof-of-concept demonstration to address the acute need for sensors that can rapidly detect emerging infectious diseases based on pathogenic genomic RNA, such as the current COVID-19 pandemic, we identified four regions of interest in the SARS-CoV-2 genome based on their uniqueness and orthogonality to other known human respiratory diseases^49,50^. We selected toehold sequences via consensus by both the LM and the CNN-based models (see “Methods”). We ligated each toehold sequence to a GFP reporter protein via a PCR reaction (see “Methods”) in order to obtain a readily measurable readout upon toehold switch induction. We evaluated predictions experimentally by measuring the fold change in the fluorescence between the ON (trigger present) and the OFF (trigger absent) states. For both the consensus model and the transfer learning CNN alone (see below, Fig. 4g, h), we found significant separation between “predicted good” and “predicted bad” sensors, consistent with our models.

### Optimizing sequences with NuSpeak and STORM

As recent results suggest synthetic riboswitches are amenable to improvement with machine learning approaches^51,52^, we sought to further optimize sequences. To that end, we constructed two optimization pipelines, coined NuSpeak (Fig. 4c) and STORM (Fig. 4d). These two frameworks differ primarily in their utility, where NuSpeak partly preserves the original trigger sequence, maintaining target fidelity, and STORM allows complete redesign of the toehold. In NuSpeak, the last 9 nucleotides of the ascending stem (positions 21–30) are randomly varied across all possible combinations (4^9^ = 262,144 variants/sequence) and each variant is evaluated with the re-trained LM and CNN-based models. Importantly, the optimized toehold maintains base pairing with the intended target for the first 21 nucleotides, and in an in silico analysis of 100 toeholds, NuSpeak significantly increases the ON/OFF ratio for a majority of the sequences (Fig. 4e). Though it appears that the top performers may deteriorate slightly during optimization, it is possible that these sequences are already located in a local fitness maximum, which may render further optimization difficult to achieve. Finally, to elucidate design rules for engineering good toehold sensors, we calculated a position-wise nucleotide frequency map for the ten best predicted variants for each parent sequence and found an enrichment of uracil in the last four positions of the switch region (Supplementary Fig. S9), echoing the results from sequence logos (Fig. 1c). This depletion of GCs in the ascending stem may suggest that the toehold un-winding in the presence of a trigger is aided by a reduction in the number of hydrogen bonds that must be overcome by the ribosome.

Given limitations in current biological circuit design processes, we additionally built a framework to rationally redesign circuit components without maintaining complementarity to a trigger sequence. We converted our initial pre-trained CNN-based model to build a Sequence-based Toehold Optimization and Redesign Model, STORM (Fig. 4d), by adapting the SeqProp method introduced in Bogard et al.^21^ Rather than using gradient descent as in the previous classification and regression tasks, we used gradient ascent to optimize sequences to meet target ON and OFF values. To evaluate the utility of STORM, we optimized the 100 worst experimental toeholds (Fig. 4f), with a significant increase in in silico predicted ON/OFF values after optimization. Saliency maps of pre- and post-optimization sequences (Supplementary Fig. S10) reveal that the model attention decreases on the first 12 nucleotides for some optimized sequences, suggesting that optimization improves toehold performance by modifying select regions.

We sought to experimentally validate these results for both platforms by optimizing the aforementioned sensors built from the SARS-CoV-2 genome. We measured an average fold-change of ~4.7 for sequences optimized with NuSpeak compared with an average fold-change of ~1.8 for the parent sequences, corresponding to ~160% improvement in sensor performance (Fig. 4g). We also applied STORM to the four “predicted bad” SARS-CoV-2 viral RNA sensors, and demonstrated experimentally that the optimized toeholds exhibited statistically significant increases in performance (Fig. 4h), with improvements in performance of 28.4×, 1.45×, 9.66×, and 2.34×, respectively, for each of the four optimized toeholds. As the sequence optimization process may create a toehold that is not complementary with the original intended biological target, we envision STORM being utilized as a valuable tool for unconstrained sequence development, such as in biological circuit component construction. As such, we built a website [https://storm-toehold.herokuapp.com] to make this prediction and redesign framework accessible to any interested researcher.

## Discussion

Given the power of modular, programmable riboregulators for diverse design applications, there is a compelling need to better integrate computational and experimental approaches. We aimed to address this prediction and design bottleneck by building STORM and NuSpeak, two deep learning frameworks that allow for the characterization, interpretation, and optimization of toehold switches and require only the RNA or DNA sequence of the trigger as input. We provide the trained models and frameworks with which to interrogate them on GitHub and a dedicated website, constituting an accessible resource for synthetic biologists who incorporate toeholds into their work.

To take advantage of a potential boost in accuracy achieved by hybrid CNN-RNN architectures^28^, we developed two complementary models. We built a CNN to function as a “motif detector” for toeholds, with several potential benefits such as the potential to capitalize on local underlying structure to train with fewer examples^46^. However, CNNs can be limited to local sequence interactions in that they only consider a fixed number of base pairs in a sliding window, with length defined by the hyperparameters of the model. With the recent successes of language models in protein modeling^53^ and promoter strength prediction^54^, as well as the development of natural language processing techniques to elucidate RNA secondary structure^55^, we implemented an language-based LSTM to further elucidate toehold sensor properties. As LSTMs consider the entire biological sequence as a whole, these model architectures are well poised to learn long-range interactions in RNA structure. In addition, while the one-hot encoding used by the CNN treats each nucleotide as an independent feature, the tokenization used as input to the LSTM implemented in our work organizes the input data into three-mers, which more closely map to codons. By using this NLP-based model in concert with convolutional architectures, we were able to reinforce the conclusions learned by each architecture.

Both models presented here offer insight into toehold design and performance prediction, underscoring the importance of designing models with interpretability in mind. Rather than treating the model as a black box and trusting its predictions, recent advancements in machine learning have emphasized the importance of understanding how and why models reach their conclusions^35,56–58^. We thus employ attention and saliency maps as valuable tools to identify possible areas of model confusion^35,56^. The CNN-based model gives us an opportunity to directly visualize the learned motifs of the network, highlighting potentially interesting biological features. Additionally, our ability to un-box the language model is a direct consequence of its QRNN architecture (a stateful architecture where the hidden state of the model is stored as a 400-dimensional vector and updated during training rather than reset in every training batch). We explore this hidden state with UMAPs to reveal that the LM has gained a meaningful understanding of toehold sequences beyond base-pair frequencies and complementarity. Synthetic biology can thus benefit from applying interpretable methods to deep learning frameworks, regardless of model type.

Furthermore, we highlighted the utility of employing transfer learning techniques that use large datasets to seed models with a set of learned weights and then fine-tune on smaller, context-specific datasets to further refine the weights. After using data ablation studies to identify how both models perform with varying dataset sizes, we found that our models maintain high performance even when trained on an order of magnitude less data than present in the Angenent-Mari et al. dataset^30^. Inspired by recent work on transfer learning for transcriptomics^59^, wherein biological patterns learned from larger datasets can be adapted to identify gene expression patterns in rare disease cohorts with few training examples, we took advantage of the models’ flexibility to train with smaller datasets by using established transfer learning techniques so as to increase prediction accuracy in different toehold contexts (e.g., fused- vs. free-trigger). Finally, we demonstrated the practical value of this transfer-learning approach by using the fine-tuned models to identify optimal sensors for the SARS-CoV-2 genome (Fig. 4g, h).

As the switch-trigger fusion used to generate a large toehold dataset (Fig. 1a) has fundamentally different effective concentrations and stoichiometries of the trigger and switch as compared to its free-trigger counterpart, we hypothesize that our fine-tuning step is important in achieving generalizability for both models. The differences between experimental setups, including altered concentrations of chaperones, ribosomes, and transcriptional co-factors, were bridged by recycling model weights. As demonstrated in our language model training, an in silico corpus is particularly useful for training stable word and sentence embeddings. This advantage can be especially pronounced in instances where the amount of training data is sparse or not uniformly sampled from the sequence space, which is often the case for nucleic acid datasets. These two transfer learning techniques of recycling weights and building an in silico corpus may offer an exciting avenue to translate results between experimental setups, especially in synthetic biology studies, which often have small or sparse datasets. Such approaches may be useful for enhancing design and biomining efforts to find synthetic biology parts based on only a few context-specific examples, augmented with recycled information from a larger dataset of related components.

In addition, we introduced two sequence optimization pipelines for divergent purposes. While the consensus model NuSpeak pipeline maintains base pairing complementarity and offers utility for pathogen sensor development, the STORM framework can be used to optimize toehold sequences for any performance constraints. Though gradient ascent is not a new concept^60^, the application of generative models to redesign linear sequences for the end goal of improving function has been recently gaining traction in protein engineering^61^. For instance, generative adversarial networks^62^ (GANs)—a modeling paradigm that simultaneously trains two competing neural networks—are being used to teach networks to produce realistic protein structure maps^63^. However, GANs remain challenging to train and define for biological tasks. By comparison, STORM readily converts our existing predictive CNN into a generative design tool without extensive re-training. In applications beyond engineered riboregulators, STORM could be used as a guide for nucleic acid modeling problems such as combinatorial biological circuit design, as well as to look inside of and augment existing prediction frameworks.

It is important to note that the tools developed here are not constrained to any single riboregulator design or dataset. Our neural network architectures can be adapted for any RNA or DNA sequence with a measurable performance, dependent only on a robust enough dataset to perform model training or re-training. Similarly, white-box tools such as attention maps and in silico mutagenesis are broadly model-agnostic, with applicability to any nucleic acid dataset. With the advent of tools to design, test, and process high-throughput biological datasets, machine learning could be exploited as a means to glean insight into biological circuit components, tools, and phenomena.

## Methods

### Toehold sequence generation

In order to define the sequences to be tested (see Angenent-Mari et al.^30^), we performed a sequence tiling on a variety of prokaryotic genomes, as well as the complete set of human transcriptional regulators. Briefly, each chosen sequence was tiled with a sequence length of 30 nucleotides and a stride of five nucleotides. Additionally, we generated a set of 10,000 random sequences of length 30, drawing each nucleotide from a uniform distribution at each position. This approach resulted in a set of 244,000 sequences to be synthesized and tested experimentally.

### Data filtering and visualization

244,000 toehold sequences were tested by Angenent-Mari et al.^30^ and the experimental data were obtained as logarithm-transformed GFP fluorescence measured at both the modified ON state (with trigger present, fused to the switch sequence) and OFF state (without trigger). Measurements were normalized and quality control was performed as indicated in Angenent-Mari et al.^30^, resulting in 91,534 sequences. All sequence logos were visualized with LogoMaker^64^. In addition, a final filtering step was applied prior to training the neural network, where sequences were split into 1000 bins for both ON and OFF distributions, and bins were down-sampled to the mean number of counts across all bins (Supplementary Fig. S11). The union of sequences from both the ON and OFF filtering stage was carried forward, resulting in 81,155 switches. Given remaining artifacts in the ON and OFF distributions, we would like to direct the reader towards experiments performed by Angenent-Mari et al.^30^, where the authors evaluate the MLP model performance with a variety of methods, including re-sampling of under-represented continuous data points to achieve a balanced distribution, and find insignificant differences in models trained without this step.

CGR representations for sequences were computed using an implementation published by Yin et al.^65^. The CGR coordinates of the set were scaled between −1 and 1, and the third (length) dimension was omitted. These coordinates were subsequently plotted using a 2D kernel density estimate with the seaborn package in order to observe macroscopic sequence patterns.

### Convolutional model architecture

The model was constructed of two convolutional layers to detect genomic motifs^33,35,66,67^. The first convolutional layer consisted of 10 filters of width 5; the second convolutional layer consisted of five filters of width 3. The filters, or weight matrices, were convolved over nucleotide channels and point-wise multiplied with the input sequence, with the magnitude of this multiplication, or activation, corresponding to the degree of similarity between the filter pattern and the input^35^. Activations from the second convolutional layer were flattened into a one-dimensional vector and fed as input to three fully connected layers with successively decreasing numbers of nodes (150, 60, 15, respectively). All layers applied the rectified linear unit nonlinearity function to node outputs^32^ and these activations were passed independently to two output layers: the ON and OFF prediction outputs, respectively. The last fully connected layer utilized linear activation to output continuous ON and OFF values. After each layer, a dropout rate^68^ of 0.3 was applied, and the ridge regression (*L*_2_ regularization) coefficient on the activations was set to 0.001 to decrease the risk of overfitting by constraining the values of the weights^32^. Errors between true and predicted ON and OFF values were computed over small batches of 128 toehold sequences per iteration using a mean squared error loss function. This loss information was backpropagated through the model, and stochastic gradient descent was used to update the weights of the model such that the disparity between model predictions and true value was minimized^32^. The Adam optimizer was used with a learning rate of 0.005 to train the model at a speed that achieved fast convergence without over- or under-shooting the optimal model fit. Weights were updated with respect to both ON prediction and OFF prediction simultaneously. Keras with a TensorFlow^69^ backend was used to construct and optimize the model.

To assess the best architecture performance and train the final model, the data were shuffled and iteratively split using tenfold cross validation; the test set per fold was further split in half to be used as validation toehold sequences to select the optimal number of training epochs. A stratified split method enabled the cross-validation to be conducted with the class imbalance preserved in each fold. A deployable model was trained on 85% of the data, with 15% held-out as validation data to enable early stopping in training.

### Hyperparameter optimization for convolutional model

Architecture design parameters were selected randomly rather than combinatorically as in a traditional grid search to enable a broader search of the architecture landscape within time and computation constraints^70^. The convolutional hyperparameters were varied to maximize the convolutional layers’ ability to learn a sufficient number of short, meaningful patterns of relevant nucleotide combinations^33,67^. To find the optimal convolutional model architecture, several hyperparameters were varied and randomly sampled (Supplementary Data 1). The number of filters tested in the first layer were 5, 10, and 15, testing each combination with filter widths of 3, 5, or 7. The number of filters tested in the second layer were 5 and 10, with filter widths of 3, 5, or 7. We also tested a select number of architectures with filter widths of 10, 15, and 20, to ensure these shorter filter widths did not restrict the model accuracy. Additional hyperparameters, such as dropout rate, *L*_2_ regularization, and Adam optimizer learning rate, were varied to curtail overfitting and modify how the space of weights was explored during training: the dropout rates tested were 0.1, 0.3, and 0.5; *L*_2_ regularization values tested were 0 and 0.0001; and learning rate values tested were 0.0005 and 0.001. For all hyperparameter combinations, *R*^2^ and Spearman correlation coefficients were evaluated across both ON and OFF values to ensure model predictions are sufficiently consistent with experimental results. Model simplicity was prioritized to avoid unnecessary complexity (i.e., more convolutional filters than needed) that did not augment the biological interpretability of the model.

Five-fold cross validation was used to train and evaluate each parameter combination. For each fold, and for ON and OFF predictions separately, *R*^2^ and Spearman’s rank correlation were calculated to estimate the generalizability of the model. The best architecture was selected by sorting the results by their combined average *R*^2^ over ON and OFF prediction, and choosing an architecture with first layer filters that would enable downstream interpretation of biologically-meaningful motifs and maximal ability to decode predictions in the context of toehold design rules.

### Language model construction

The LM was trained on an in silico corpus of 4 million synthetic toehold sequences. We generated random 30 nucleotide sequences by sampling each nucleotide with uniform probability, and filled in the remainder of the 59 nucleotide sequence with the basic set of toehold structural rules (Fig. 1a, Supplementary Table S1). These in silico sequences were sampled from a vast sequence space (4^30^), and thus had little collinearity (Supplementary Fig. S12), as evidenced by the fact that the distribution of pairwise edit distances are approximately centered at 22 nucleotides and 35 nucleotides for the switch region and complete toehold respectively.

The basal NuSpeak architecture was derived from the PyTorch^71^ implementation of ULMFit^72^ provided by fastai [https://docs.fast.ai/]. Custom tokenization and vocabulary functions were written using the fastai NLP wrapper in order to process genomic sequences as opposed to human languages. In brief, our modified ULMFit consists of a four-layer QRNN^39,40^ with 1552 hidden activations in each layer, sandwiched between an embedding layer and a decoder layer. The LM classifier, likewise, consists of an embedding layer, a four-layer QRNN, and a linear classifier head, in lieu of the decoder. As suggested in Merity et al.^73^, we found that tying the weights between the embedding and output decoder layer reduced LM perplexity after training. Dropouts for the output, hidden, input, and embedding layers were set to 0.15, 0.20, 0.30, and 0.05, respectively. Weight-decay dropout was set to 0.25 across all layers and weight-decay was set to 0.1 to provide strong *L*_2_ regularization during LM training. Most of these parameters were kept the same when training the LM classifier, with the exception of the output dropout, which was set to 0.5. Both the LM encoder and classifier were trained using the Ranger optimizer [https://github.com/lessw2020/Ranger-Deep-Learning-Optimizer], which synergistically combines a Rectified Adam with the LookAhead optimization approach as described in Zhang et al.^74^. In addition, all models were trained using a batch size of 128 with back-propagation-through-time set to 60 in mixed-precision so as to maximize generalizability. A cosine learning rate schedule^75^ was used during LM training for a total of 15 epochs, while a label smoothing cross-entropy loss^76^ was used while training the classifier. Furthermore, to obtain a direction-agnostic view of toehold sequences, forwards and backwards models were separately trained and subsequently averaged during model prediction on the held-out test sets.

### Language model evaluation

A random sampling of 5000 sequences from the experimental dataset were fed into the pre-trained LM encoder, which was trained to identify the top 25% vs the bottom 75% of toeholds. The 400-dimensional hidden state of the LM encoder was concatenated and averaged across all tokens for each input sequence in the randomly selected sample. For comparison, shuffled and scrambled input sequences, as well as random 59 nucleotide sequences, were also fed into the pre-trained toehold LM encoder. The shuffled input sequence was generated by shuffling the order of tokens in a tokenized input sequence and provides insight on the extent to which the model learns k-mer frequencies. Meanwhile, the scrambled input sequence was generated by first scrambling the real input sequence prior to tokenization, and offers insight on the extent to which the model learns nucleotide frequencies. We focused exclusively on classification tasks given that the architectures developed thus far in NLP are tailored for classification^77^, though we note it would be possible to add a linear output layer for regression on top of the model discussed here.

In addition, to assess the impact of language model pre-training for the classification task, we built and trained a naïve classifier which also consists of an embedding layer, followed by a four-layer QRNN and a linear classifier head. Tokenized sequences from the experimental dataset were then directly fed into the naïve model without pre-training on in silico sequences. We observed that pre-training the LM on the in silico set of 4 million toeholds significantly stabilizes the model’s word embeddings and yields statistically better performance on held-out test sets. To evaluate attention of the model, the fastai intrinsic attention function was used [https://github.com/fastai/fastai1/blob/master/fastai/text/interpret.py], which calculates the contribution of each k-mer in a sequence for the resulting gradient, given a classification prediction.

### Language model comparison

We compared the language model to other NLP-based models. To compute tf-idf matrices, input sequences were tokenized in the same manner as for the language model, and tf-idf models were constructed using unigrams and bi-grams of three-mers (scikit-learn). The Naïve-Bayes, Logistic Regression, and Random Forest algorithms were used from off-the-shelf implementations provided in scikit-learn, along with a grid search used for optimizing their respective hyperparameters. To compute skip-gram embeddings, input sequences were again tokenized into three-mers with stride 3, and a word2vec algorithm^78^ [https://radimrehurek.com/gensim/models/word2vec.html] was used to learn contextual word embeddings across the entire experimental dataset. The pre-trained word embeddings were then used as inputs to an array of deep learning architectures as shown in Supplementary Fig. S7, connected to a simple classifier head. Deep learning architectures were built using off-the-shelf implementations provided by Keras and Tensorflow^69^. The self-attention layer was sourced from Keras [https://github.com/CyberZHG/keras-self-attention] for the attention-based architecture. All model specific hyperparameter details are enumerated in the source code files.

### Saliency maps

Saliency maps were generated to visualize which positions and nucleotides mattered most towards high ON and low OFF model predictions^35,56,57^. The keras-vis package was used to analyze how small changes in a given input toehold sequence change the model’s output predictions. The gradients were computed to highlight changes to the input sequence that produce large changes in the output predictions, revealing which positions in the toehold sequence were prioritized the most when predicting ON and OFF values. A saliency (i.e., an “importance score”) for each position and each nucleotide at that position was calculated by summing the gradients across all positions and nucleotides for each toehold. This saliency was normalized by the number of times a given nucleotide appeared at that position to control for more frequent nucleotides.

### Consensus model

For the consensus model, we first used transfer learning to freeze weights of both the NLP-based and CNN-based model, and re-trained both on a set of 168 toehold sequences from Green et al.^8^ for fine-tuning. For the CNN-based model, we modified the last output layer to output one value—the combined ON/OFF ratio alone—to be compatible with the normalized ON values reported in Green et al.^8^ An in silico external validation was conducted by comparing the ranks of the 24 Series A toeholds tested in Pardee et al.^10^ with the ranks predicted by the original model, a model trained on Green et al. sequences^8^ alone, and the transfer learning models.

We then implemented a consensus algorithm to pick out toehold sequences that were both classified as good with high probability by the LM, and predicted to yield high ON/OFF ratios by the CNN model. In brief, our simplex routine consisted of rank ordering by the geometric mean of the classification probability score and the predicted ON/OFF ratio score. We used the geometric mean over the arithmetic mean since it is more robust to extremes in either model. Of the sequences that passed through both models, eight “predicted good” and eight “predicted bad” were chosen to be experimentally validated. These eight test sequences were chosen at random amongst the list of sequences that passed both models. Experimentally tested sequences are detailed in Supplementary Table S2.

We separately chose five “predicted good” and four “predicted bad” sequences ranked by the CNN-based model as comparison, restricting our set to nine toeholds due to experimental limitations. We generated the list of all possible 25,220 sequences in the SARS-CoV-2 regions of interest and again took advantage of the consensus approach, and we ran each sequence through the original CNN, the transfer learning model, and the model trained on Green et al. sequences^8^ alone. Although the model trained on Green et al. sequences^8^ alone is likely underfit given the sparse data, we had an abundance of sensors that passed the filtering criteria from the two previously described models and thus sought to further filter these top predictions. We then picked the top five sequences and four of the bottom six sequences, filtered by those that had the least negative MFE as predicted by NUPACK. Experimentally tested sequences are detailed in Supplementary Table S3.

### Optimization methods

To optimize sequences using the consensus model (aka NuSpeak), we generated all possible variants by modifying the last nine nucleotides of each “predicted bad” 30 nucleotide sequence validated with the consensus model. The variants for each parent sequence were fed back into the pre-trained classifier to determine a classification probability score for each sequence, after which all sequences were fed into the pre-trained CNN regressor to predict the ON/OFF ratio. Our simplex routine was then used to identify variants that passed both sets of models and we selected eight optimized sequences for experimental validation.

To optimize sequences using the STORM, we converted our CNN-based predictive pipeline to redesign poorly performing toehold switches via gradient ascent. The 20 toeholds with the lowest ON/OFF ratio were one-hot encoded and fed as inputs to the static model. A target ON/OFF value of 1 was set and supplied to SeqProp, an open-source python package that enables streamlined development of gradient ascent pipelines for genomic and RNA biology applications^21^. Toehold design constraints were ignored during optimization, as the entire 59 nucleotide sequence was allowed to vary freely.

After optimization, we “fixed” each sequence such that the modified toehold switch contained the conserved sequences and base pairing within the hairpin was preserved. We also offer the ability to incorporate these base pairing constraints in the loss function itself, but found that the model sometimes settled into a local minimum and did not optimize effectively. At each iteration, the ON/OFF value of the initial toehold sequence were predicted and the difference between the predicted values and target values was computed. This discrepancy between predicted and target values was then propagated back through the model to update the input sequence in the direction that decreased the difference between the predicted ON/OFF value and the target. The updated toehold position weight matrix was used as input to the next round of optimization, and at the last round of iteration, the final sequence was composed of nucleotides with the highest probabilities in the position weight matrix. At each iteration, there is a balance between exploration and exploitation with an entropy parameter, as in some Bayesian optimization frameworks. Each sensor went through five rounds of optimization, and the resulting sequence with the highest ON/OFF value was chosen. Improvements in performance were quantified as the fold-change of the ON/OFF fold-changes between pre- and post-optimization.

### Toehold switch validation

Toehold switch reactions were validated in cell-free protein synthesis systems as described in Pardee et al.^9^ In brief, switches were individually ordered as 109nt Ultramer DNA oligonucleotides (IDT) consisting of a T7 promoter, 59 nucleotide switch region, common linker, and the first 5 nucleotides of a GFP sequence. Ultramers were added to a GFP gene (GFPmut3b-ASV with T7 terminator) through PCR amplification using Q5 high-fidelity polymerase (NEB). Resultant amplicons were treated with DpnI (NEB) to remove residual template DNA, and subsequently purified using a MinElute PCR purification Kit (Qiagen). Separately, triggers were synthesized from oligos consisting of antisense T7 promoter and antisense trigger sequence annealed to the sense strand of the T7 promoter using the HiScribe T7 Quick High Yield RNA Synthesis Kit (NEB). Reactions were incubated for 16 h at 37 °C, treated with DNaseI (NEB), and purified using the RNA Clean & Concentrator-25 kit (Zymo Research). Cell-free toehold switch reactions were performed using the PURExpress In Vitro Protein Synthesis Kit (NEB). Each reaction contained NEB Solution A (40% v/v), NEB Solution B (30% v/v), Murine RNase inhibitor (0.5% v/v; NEB), purified toehold switch PCR product (5 nM), and either no-trigger RNA (OFF state) or 10μM of trigger RNA (ON state). All reactions contained 5μL total volume and were carried out in triplicate within 384-well plates (Corning 3544) at 37 °C. GFP expression (485 nm excitation, 520 nm emission) was monitored on a plate reader (Molecular Devices SpectraMax M3) every 5 min for 150 min. OFF and ON values were taken as endpoint GFP fluorescence from reactions without trigger and with trigger, respectively. The GFP sequence used in this study can be accessed on Benchling [https://benchling.com/s/seq-Su3ovggGNrVRzsPxjAeA].

### Reporting summary

Further information on research design is available in the Nature Research Reporting Summary linked to this article.

## Supplementary information

Supplementary Information

Reporting Summary

Supplementary Data 1

Description of Additional Supplementary Files

Source Data File

## Data Availability

Complete toehold screening data is provided with Angenent-Mari et al. as well as in the data folder of a public GitHub repository engineered-riboregulator-ML [github.com/midas-wyss/engineered-riboregulator-ML/tree/master/data]. Any other relevant data are available from the authors upon reasonable request. Source data are provided with this paper.

## Code availability

Python code for neural net construction, training, and prediction can be found at this public GitHub repository engineered-riboregulator-ML [github.com/midas-wyss/engineered-riboregulator-ML].
