# Supplementary material for: Sequence-to-function deep learning frameworks for engineered riboregulators
Source: Nat Commun. 2020 Oct 7;11:5058. doi: 10.1038/s41467-020-18676-2 (PMC7541510; doi:10.1038/s41467-020-18676-2)
Supplement: Supplementary file 1 — Supplementary Information [file 41467_2020_18676_MOESM1_ESM.pdf]

Supplementary Information

Sequence-to-function deep learning frameworks for engineered riboregulators

Valeri, Collins, Ramesh, et al.

<u>Supplementary Figures</u>

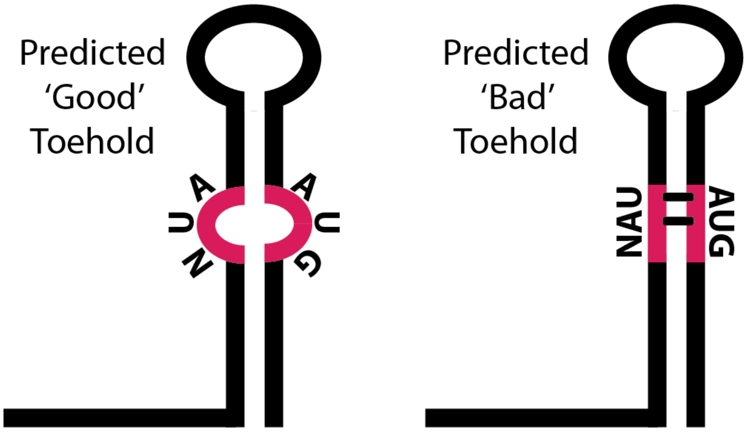

Supplementary Figure 1. **NUA is over-represented in the three-nucleotide bulge opposite the start codon.** High-performing toeholds have an over-representation of NUA in positions 22 – 24, the three-nucleotide bulge opposite the start codon, suggesting that top-performing sequences do not hybridize with the start codon.

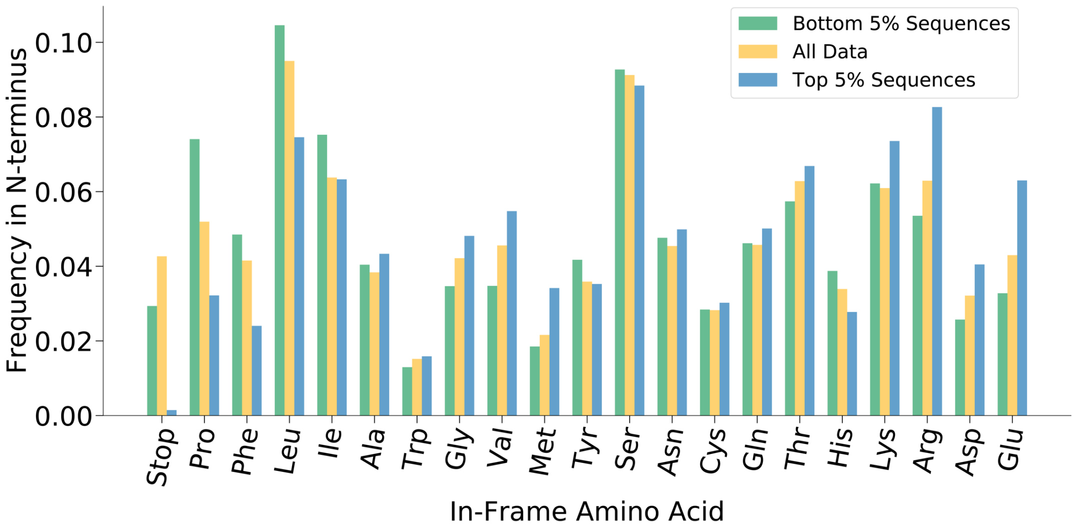

Supplementary Figure 2. **High-performing and poorly-performing switches show over-representation for specific types of amino acids in the N-terminus.** Raw frequencies of each in-frame amino acid were calculated for all sequences (yellow, $N = 91{,}534$), as well as for the top 5% (blue, $N = 4{,}577$) and bottom 5% (green, $N = 4{,}577$) of sequences when stratified according to experimental ON/OFF ratios. Under-representation of stop codons, proline, and phenylalanine is evident in top sequences, while top sequences seem to prefer charged amino acids in the N-terminus.

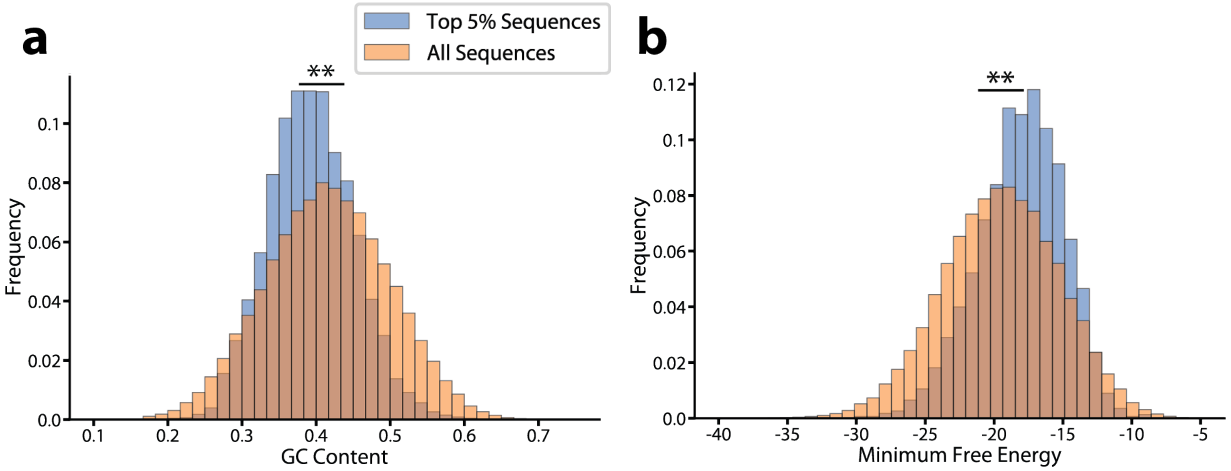

Supplementary Figure 3. **Biophysical properties are not adequate to predict toehold performance on their own.** a) GC content distributions and b) switch minimum free energy estimates according to NUPACK were calculated for all sequences (orange, *N* = 91,534) and the top 5% of sequences (blue, *N* = 4,577), with clear differences in distributions for GC content ($p = 1.21 \times 10^{-87}$) and minimum free energy ($p = 2.63 \times 10^{-132}$). However, given the large overlaps in distributions, these properties are not solely predictive of toehold performance on their own. All tests are two-tailed Mann-Whitney U tests.

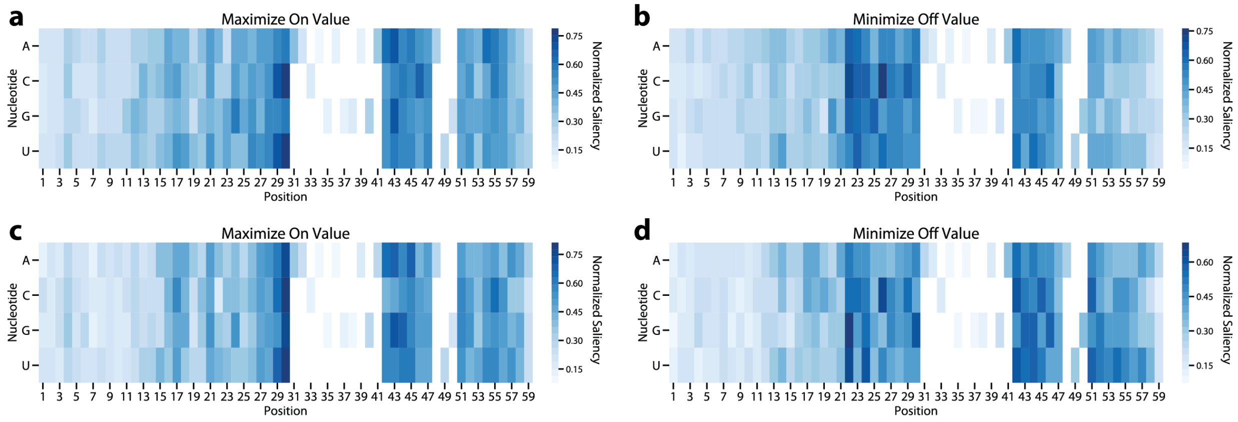

Supplementary Figure 4. **Saliency maps are not markedly different for toehold switches with good and bad performance.** a) To ensure that the saliency maps did not change dramatically based on which sequences were tested, saliency was calculated on a set of 100 'good' switches using the convolutional neural network model. Saliency maps were generated to both maximize the ON prediction and b) minimize the OFF prediction. c) Saliency maps were also generated for 100 'bad' switches, both maximizing the ON prediction and d) minimizing the OFF prediction. Switches were randomly chosen from top and bottom performing toeholds based on experimental ON/OFF ratios. All saliency maps indicate the importance of the regions immediately surrounding the Shine-Dalgarno sequence, while de-emphasizing the importance of the first 12 nucleotides.

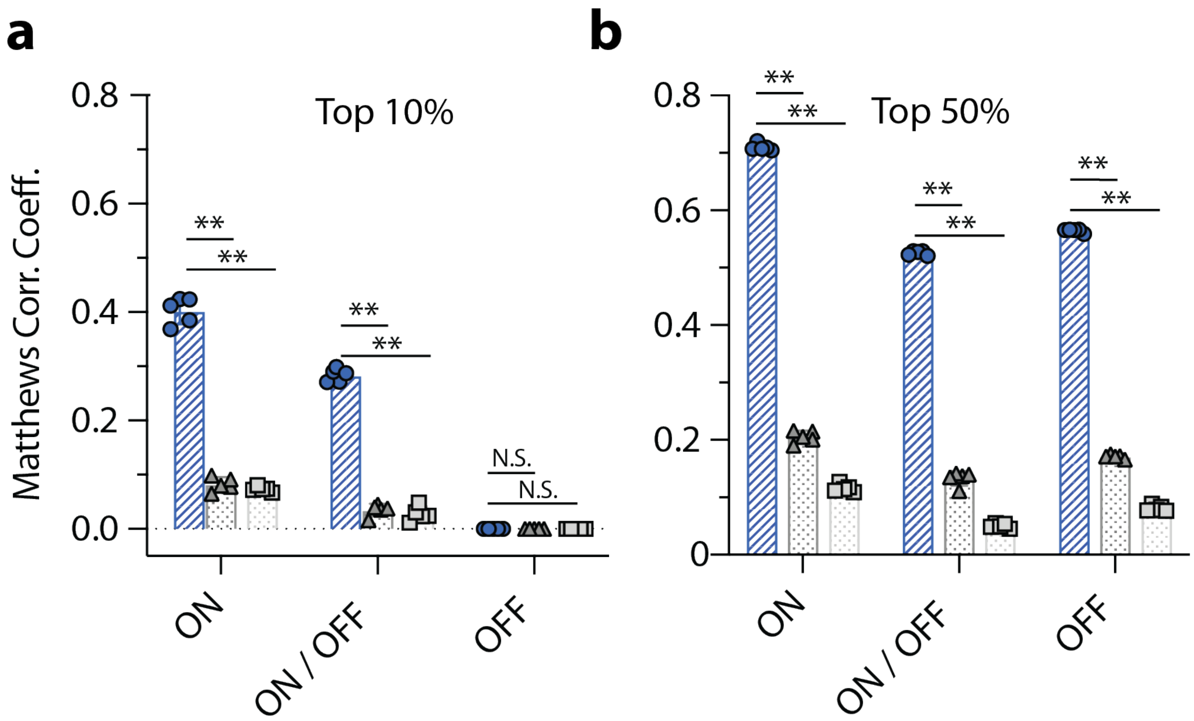

Supplementary Figure 5. **Language model performance varies based on the ON/OFF threshold used for classification.** Three different thresholds were explored for classification, each with five-fold cross validation: a) a 10%/90% threshold, a 25%/75% threshold (Fig. 3A), and b) a 50%/50% threshold. For all thresholds, the language model performs significantly worse on shuffled (grey triangle) or scrambled toehold (grey square) and only accurately classifies real toeholds (blue circle), as evaluated by Matthews Correlation Coefficient (MCC). Additionally, classification of ON values alone outperforms classification of ON/OFF values and OFF values alone. We chose the 25%/75% threshold because the ON/OFF ratio is an internally normalized performance metric and the fluorescence data were sorted into four bins. a) For the 10% classification threshold, ON MCC is higher for real toeholds than shuffled ($p = 5.79x10^{-9}$) or scrambled ($p = 2.05x10^{-9}$) sequences. ON/OFF classification is also more accurate for real toeholds than shuffled ($p = 6.24x10^{-10}$) or scrambled ($p = 1.10x10^{-9}$). OFF MCCs are all zero at this threshold. b) For the 50% classification threshold, ON MCC is higher for real toeholds than shuffled ($p = 2.60x10^{-13}$) or scrambled ($p = 6.78x10^{-15}$) sequences. ON/OFF classification is also more accurate for real toeholds than shuffled ($p = 2.76x10^{-12}$) or scrambled ($p = 6.15x10^{-16}$). Finally, OFF MCC is higher for real toeholds than shuffled ($p = 4.55x10^{-16}$) or scrambled ($p = 1.01x10^{-15}$) sequences. For all panels, $N = 5$ trials, error bars represent mean +/- standard deviation, and all tests are two-tailed t-tests.

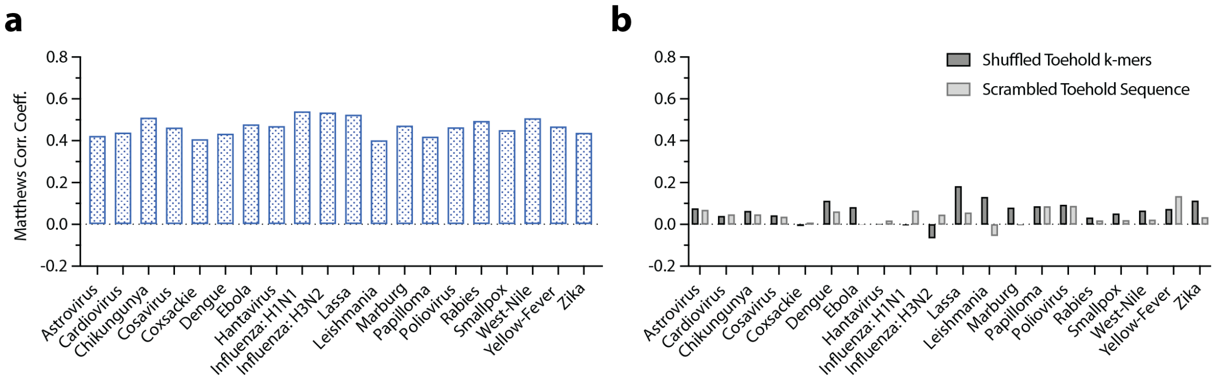

Supplementary Figure 6. **Language model-based classification is generalizable to viral genomes not used in the training stage.** a) The language model was trained on toeholds tiled from human transcription factors and generated by random sequences, while all viral genomes were excluded during the training step. The model was then evaluated on its predictions on each viral genome. b) When comparing these viral toeholds with scrambled (light grey) or shuffled (dark grey) toeholds, the model can generalize to toeholds from unseen genomes.

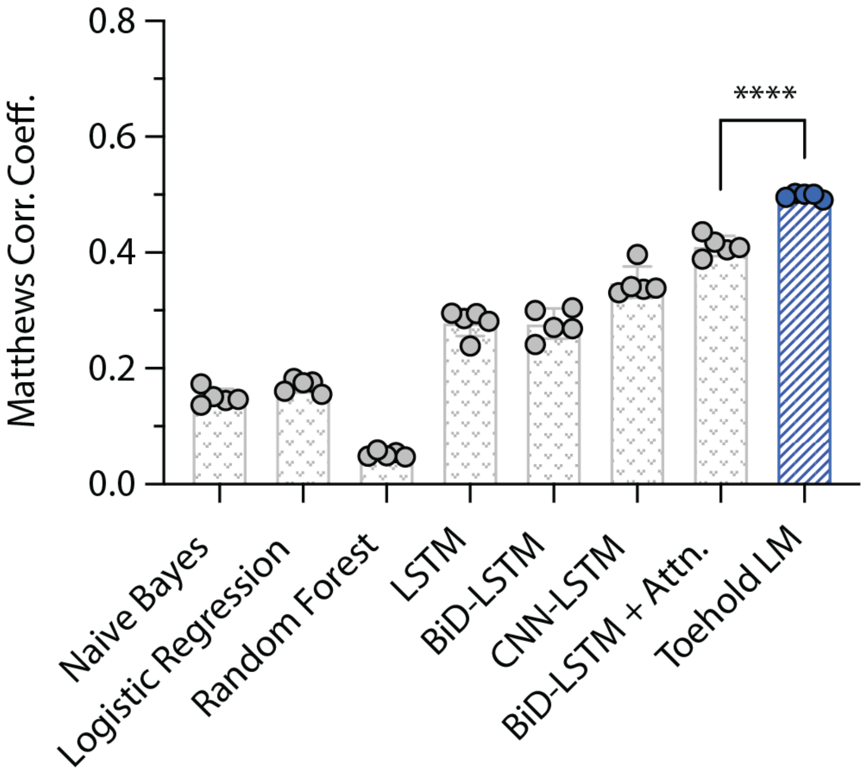

Supplementary Figure 7. **Language model-based classification outperforms off-the-shelf classification models.** We compared our language model (LM) to several off-the-shelf classification models, each in a five-fold cross validation setting. The toehold language model significantly improved upon the bidirectional LSTM (Long Short-Term Memory) model with an attention layer when classification was evaluated with Matthews Correlation Coefficient ($p = 5.01x10^{-6}$). Error bars represent mean +/- standard deviation for $N = 5$ cross validation folds and all tests are two-tailed t-tests.

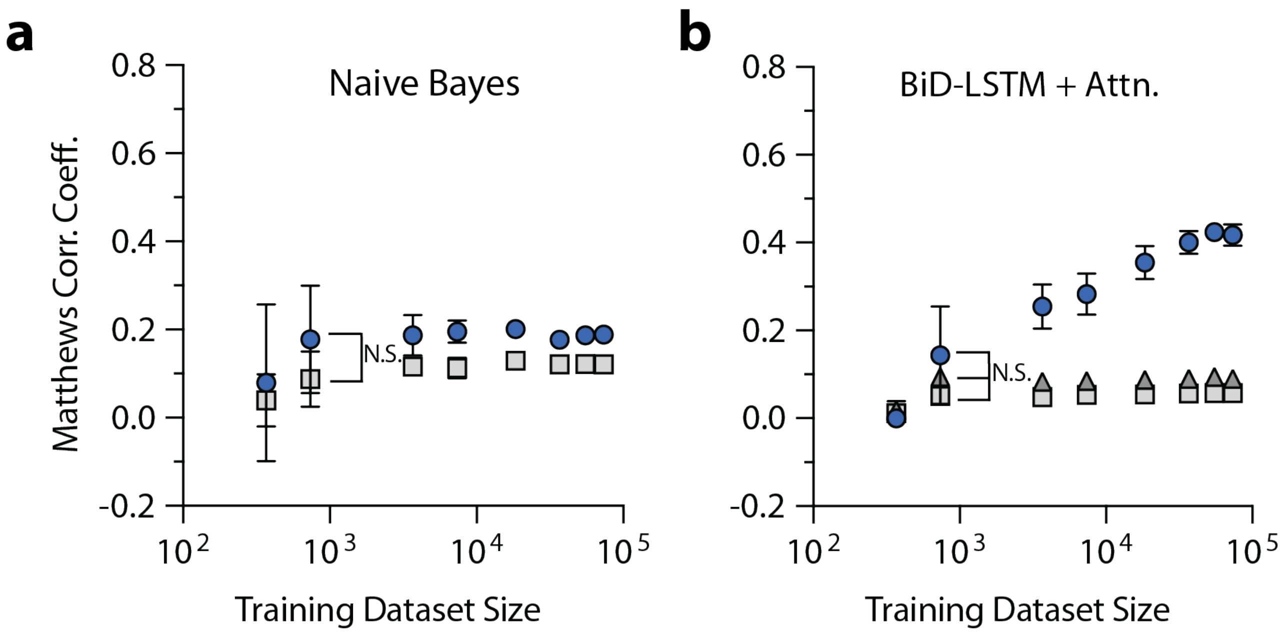

Supplementary Figure 8. **Standard models are less robust to data ablation.** a) To investigate how other standard models performed with less data, we performed data ablation trials on both a Naïve Bayes model as well as b) the bidirectional LSTM (Long Short-Term Memory model) plus attention layer. For all types of models, performance is significantly worse on shuffled (grey triangle) or scrambled toeholds (grey square) than for real toeholds (blue circle). Both the Naïve Bayes and BiD-LSTM fail to achieve meaningfully different performance for real and scrambled toeholds with 736 training examples ($p = 0.182$, $p = 0.093$). The BiD-LSTM fails to achieve meaningfully different performance for real and shuffled sequences ($p = 0.184$) with 736 training examples. Both models achieve a lower maximum Matthews Correlation Coefficient than the language model described in Fig. 3. For all panels, error bars represent mean +/- standard deviation of 5 trials and all tests are two-tailed t-tests.

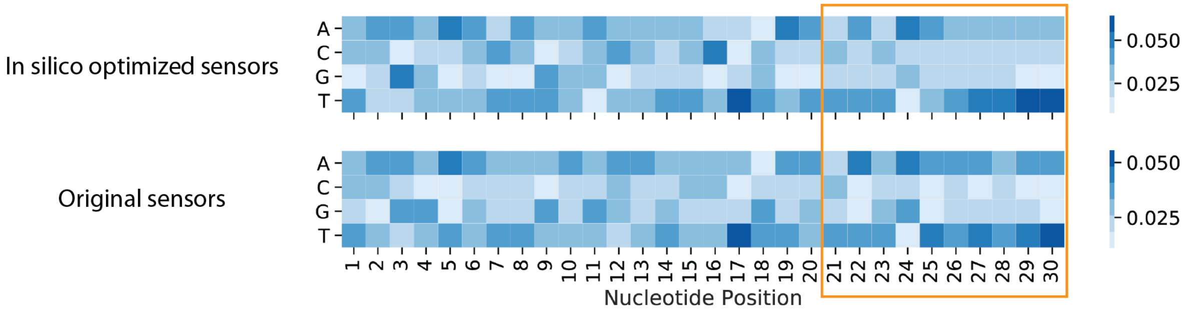

Supplementary Figure 9. **Frequency maps of sensors pre- and post-optimization via NuSpeak show GC under-enrichment in the ascending toehold stem.** We mapped the nucleotide frequencies of 100 sequences before and after optimization to identify patterns in the optimization process. While the first 21 nucleotides are fixed during optimization, variation in the last nine nucleotides is evident. As in Fig. 1C, top sequences show an under-enrichment of guanine and cytosine in the ascending stem, possibly as a result of more readily un-winding in the ON state with trigger present.

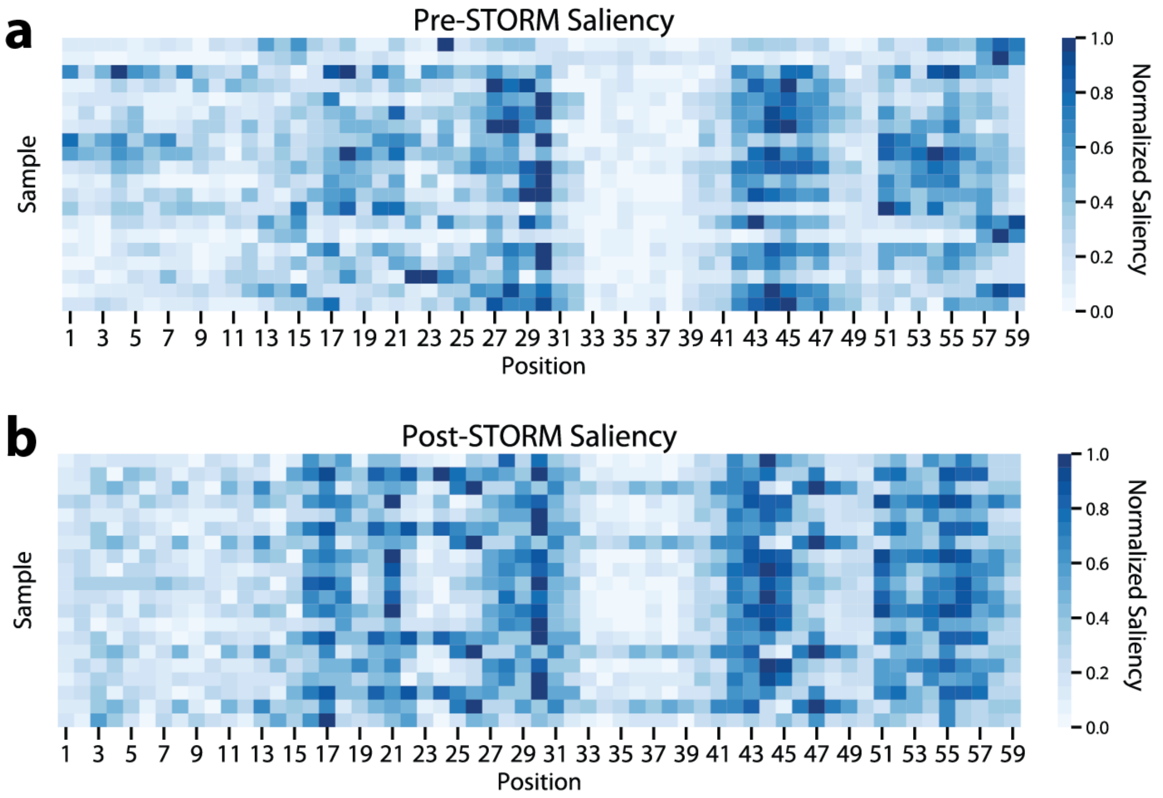

Supplementary Figure 10. **Differential attention to first twelve nucleotides in saliency maps for gradient ascent sequences pre- and post-optimization.** a) Given a set of 20 bad toeholds that were optimized via our gradient ascent framework, we sought to understand how the model's focus changed when predicting performance before and after gradient ascent. Saliency maps to illustrate the parts of the sequence that maximized the ON prediction were generated for the pre-optimization sequences and b) post-optimization sequences. For several of the 20 sequences sampled, the model pays less attention to the first 12 nucleotides after optimization.

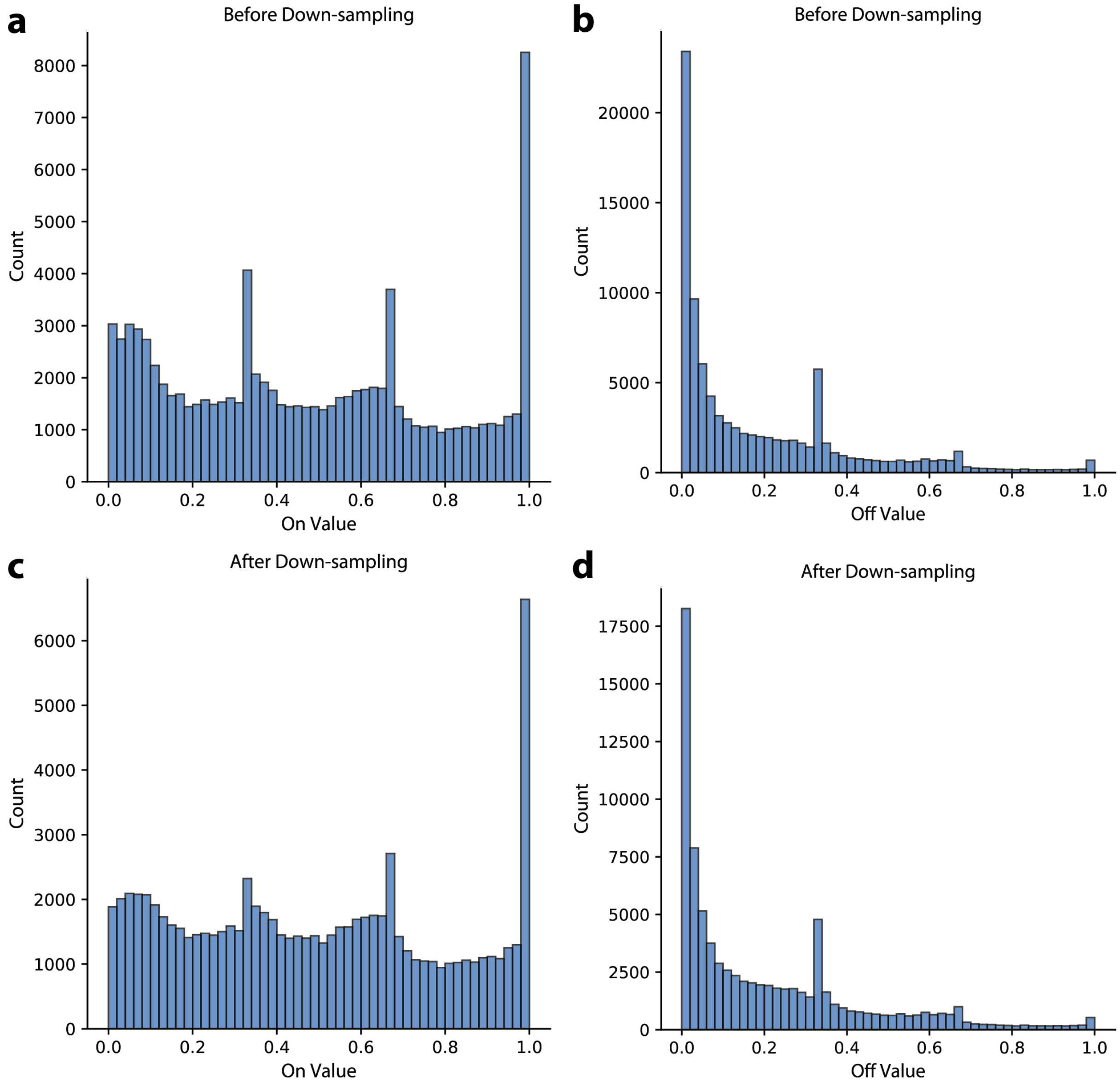

Supplementary Figure 11. **Down-sampling preserves the ON and OFF value distributions, while reducing experimental artifacts.** a) Given the abnormally high counts observed at several ON and b) OFF values, the ON and OFF distributions were trimmed such that the number of sequences in each of 1000 evenly-spaced bins was reduced to the mean number of sequences over all bins. We took the union of the sequences that passed either ON or OFF filtering for a total of 81,155 sequences, showing here the resulting c) ON and d) OFF value distributions.

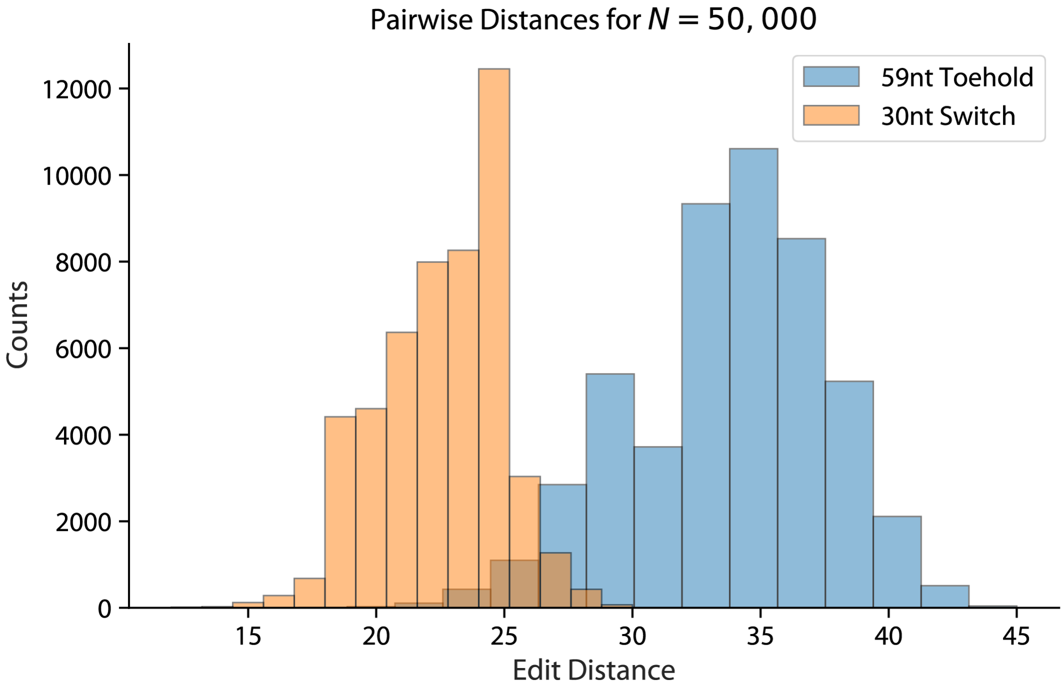

Supplementary Figure 12. **Pairwise edit distances between sets of random toeholds show little collinearity in synthetic sequences.** To demonstrate that the synthetically generated toeholds were sufficiently different from each other, we calculated pairwise edit distances for ten sets of 5,000 toeholds for both the 30 nucleotide variable switch region (orange) and the corresponding 59 nucleotide toehold (blue). The peak for both distributions in the case of highly similar sequences would be close to 0. These distributions are upper bounded at 30 nucleotides and 45 nucleotides for the switch and toehold, respectively, due to conserved regions in the toehold. We observe the prevalence of edit distances between 23 and 25 nucleotides for the switch sequences, and between 35 and 40 for the toeholds, which reinforce the expectation of dissimilarity between sequences and consequent non-collinearity.

Supplementary Tables

Component	Start Position	End Position	Length (nts)	Complementary to
Unstructured Region	1	12	12	Trigger (19-30)
Ascending Stem 1	13	21	9	Trigger (10-18), Descending Stem 2
Bulge Opposite AUG	22	24	3	
Ascending Stem 2	25	30	6	Trigger (1-6), Descending Stem 1
Shine-Dalgarno / Ribosome Binding Site (RBS)	31	41	11	
Descending Stem 1	42	47	6	Ascending Stem 2
AUG	48	50	3	
Descending Stem 2	51	59	9	Ascending Stem 1

Supplementary Table 1. **Toehold switch anatomy consists of an unstructured region followed by a hairpin.** The hairpin stem relies on complementary nucleotides to maintain secondary structure. The loop of the hairpin contains the Shine-Dalgarno sequence, which becomes accessible to a ribosome when the 30-nucleotide trigger binds to the complementary switch region and melts the hairpin.

Sequence Type	Original	Optimized
predicted good	GTATTGTTATAGCGGCCTTCTGTAAAACACA ACAGAGGAGAGTGTTTATGAGAAGGCCG	
predicted good	ATACTGTGAAATTCCATCTAGTATTGTTATA ACAGAGGAGAATAACAATGCTAGATGGA	
predicted good	AGAAGAATCACCAGGAGTCAAATAACTTCT AACAGAGGAGAAGAAGTATGTTGACTCCT	
predicted good	TTTTAATAGAAAAGTCCTAGGTTGAAGATAA ACAGAGGAGATATCTTATGCCTAGGACT	
predicted good	GTTTGAGATTAGACTTCCTAAACAATCTATA ACAGAGGAGAATAGATATGTTAGGAAGT	GTTTGAGATTAGACTTCCTAATTATTCTCTA ACAGAGGAGAAGAGAAATGTTAGGAAGT
predicted good	AAGGTTTGAGATTAGACTTCCTAAACAATCA ACAGAGGAGAGATTGTATGGGAAGTCTA	AAGGTTTGAGATTAGACTTCCTAAGCTCTA AACAGAGGAGATAGAGCATGGGAAGTCTA
predicted good	CTGATAACTAGCGCATATACCTGCACCAATA ACAGAGGAGAATTGGTATGGGTATATGC	CTGATAACTAGCGCATATACCTCATCTTCT AACAGAGGAGAAGAAGAATGGGTATATGC
predicted good	CGAGGAGAATTAGTCTGAGTCTGATAACTAA ACAGAGGAGATAGTTAATGGACTCAGAC	CGAGGAGAATTAGTCTGAGTCATAATCTTA AACAGAGGAGATAAGATATGGACTCAGAC
predicted bad	TTATAGCGGCCTTCTGTAAAACACGCACAGA ACAGAGGAGACTGTGCATGTTTTACAGA	
predicted bad	AGCTGTCCAACCTGAAGAAGAATCACCAGG AACAGAGGAGACCTGGTATGTCTTCTTCA	
predicted bad	CTTCAACACCATTACAAGGTGTGCTACCGGA ACAGAGGAGACCGGTAATGCACCTTGTA	
predicted bad	CTAGCTACACTACGTGCCCGCCGAGGAGAA AACAGAGGAGATTCTCCATGGCGGGCACG	
predicted bad	GAAATTCCATCTAGTATTGTTATAGCGGCCA ACAGAGGAGAGGCCGCATGAACAATACT	GAAATTCCATCTAGTATTGTTTCACCTTCTA ACAGAGGAGAAGAAGGATGAACAATACT
predicted bad	CTGTCCAACCTGAAGAAGAATCACCAGGAG AACAGAGGAGACTCCTGATGATTCTTCTT	CTGTCCAACCTGAAGAAGAATCTAATCTTA AACAGAGGAGATAAGATATGATTCTTCTT
predicted bad	AATATCTCTCTCAAAAGGTTTGAGATTAGAA ACAGAGGAGATCTAATATGAAACCTTTT	AATATCTCTCTCAAAAGGTTTTCATCTTCTA ACAGAGGAGAAGAAGAATGAAACCTTTT
predicted bad	CTGAGTCTGATAACTAGCGCATATACCTGCA ACAGAGGAGAGCAGGTATGTGCGCTAGT	CTGAGTCTGATAACTAGCGCAATAATACTA AACAGAGGAGATAGTATATGTGCGCTAGT

Supplementary Table 2. **Original and optimized toeholds predicted by the language model and tested experimentally.** Eight 'predicted good' and eight 'predicted bad' toeholds were synthesized and tested experimentally. We optimized four 'predicted good' toeholds and four 'predicted bad' toeholds via NuSpeak to achieve a higher ON/OFF ratio.

Sequence Type	Original	Optimized
predicted good	CTTAAGCTTAAGTACACAATTTTGCATAGAA ACAGAGGAGATCTATGATGAATTGTGTA	
predicted good	TGCCACCAACACCCAACAATTTAATGTTGAA ACAGAGGAGATCAACAATGAATTGTTGG	
predicted good	AATTTTGAAGGTCACACTTTTCTAATAGCAA ACAGAGGAGATGCTATATGAAAAGTGTG	
predicted good	ACTTCTACTAAGCCACAAGTGCCATCTTTAA ACAGAGGAGATAAAGAATGCACTTGTGG	
predicted good	CACTAGTGTAGGTGCACTTAATGGCATTACA ACAGAGGAGAGTAATGATGTTAAGTGCA	
predicted bad	TGTATAAACCCACAAATGTAAGTGAAAAAA AACAGAGGAGATTTTTTATGTTACATTTG	TCCTTCGGCATCTACATCTATATAAAACGA AACAGAGGAGATCGTTTATGATAGATGTA
predicted bad	AATGTCCACACCCAAATTATTGAGTATTTTA ACAGAGGAGAAAAATAATGAATAATTTG	CTCATTATTATCTGCTGGTCTTCCCCTCCAA ACAGAGGAGATGGAGGATGAGACCAGCA
predicted bad	GTTGTTTAATCCTTTAATAAAGTATAAATAA ACAGAGGAGATATTTAATGTTTATTAAA	GATGGTTAAAACCCGACAACTATAGATCTA AACAGAGGAGATAGATCATGAGTTGTCGG
predicted bad	ATCAAAGTGTCCCTTATTTACAACATTAAAA ACAGAGGAGATTTAATATGGTAAATAAG	TTCATTATTATCTGCTGCTCTTCCCCTCCAA ACAGAGGAGATGGAGGATGAGAGCAGCA

Supplementary Table 3. **Original and optimized toeholds predicted by the convolutional neural network-based model and tested experimentally.** Five ‘predicted good’ and four ‘predicted bad’ toeholds were synthesized and tested experimentally. We optimized these four ‘predicted bad’ toeholds via the Sequence-based Toehold Optimization and Redesign Model (STORM) to achieve a higher ON/OFF ratio.
