# Supplementary material for: Sequence-to-function deep learning frameworks for engineered riboregulators
Source: Nat Commun. 2020 Oct 7;11:5058. doi: 10.1038/s41467-020-18676-2 (PMC7541510; doi:10.1038/s41467-020-18676-2)
Supplement: Supplementary file 2 — Reporting Summary [file 41467_2020_18676_MOESM2_ESM.pdf]

nature research

Corresponding author(s): Diogo M. Camacho

Last updated by author(s): Sep 1, 2020

Reporting Summary

Nature Research wishes to improve the reproducibility of the work that we publish. This form provides structure for consistency and transparency in reporting. For further information on Nature Research policies, see our Editorial Policies and the Editorial Policy Checklist.

Statistics

For all statistical analyses, confirm that the following items are present in the figure legend, table legend, main text, or Methods section.

n/a	Confirmed	
☐	☒	The exact sample size (n) for each experimental group/condition, given as a discrete number and unit of measurement
☐	☒	A statement on whether measurements were taken from distinct samples or whether the same sample was measured repeatedly
☐	☒	The statistical test(s) used AND whether they are one- or two-sided<br>*Only common tests should be described solely by name; describe more complex techniques in the Methods section.*
☐	☒	A description of all covariates tested
☐	☒	A description of any assumptions or corrections, such as tests of normality and adjustment for multiple comparisons
☐	☒	A full description of the statistical parameters including central tendency (e.g. means) or other basic estimates (e.g. regression coefficient) AND variation (e.g. standard deviation) or associated estimates of uncertainty (e.g. confidence intervals)
☐	☒	For null hypothesis testing, the test statistic (e.g. F, t, r) with confidence intervals, effect sizes, degrees of freedom and P value noted<br>*Give P values as exact values whenever suitable.*
☒	☐	For Bayesian analysis, information on the choice of priors and Markov chain Monte Carlo settings
☐	☒	For hierarchical and complex designs, identification of the appropriate level for tests and full reporting of outcomes
☐	☒	Estimates of effect sizes (e.g. Cohen's d, Pearson's r), indicating how they were calculated

Our web collection on statistics for biologists contains articles on many of the points above.

Software and code

Policy information about availability of computer code

Data collection	N/A
Data analysis	Python code for neural net construction, training, and prediction can be found at this public GitHub repository engineered-riboregulator-ML [github.com/midas-wyss/engineered-riboregulator-ML]. Included in this repository is a requirements.txt file which lists all software and versions used in this study.

For manuscripts utilizing custom algorithms or software that are central to the research but not yet described in published literature, software must be made available to editors and reviewers. We strongly encourage code deposition in a community repository (e.g. GitHub). See the Nature Research guidelines for submitting code & software for further information.

Data

Policy information about availability of data

All manuscripts must include a data availability statement. This statement should provide the following information, where applicable:

- Accession codes, unique identifiers, or web links for publicly available datasets
- A list of figures that have associated raw data
- A description of any restrictions on data availability

Source data for Figures 4G and 4H are provided with this paper. Complete toehold screening data is provided with Angenent-Mari et al. as well as in the data folder of a public GitHub repository engineered-riboregulator-ML [github.com/midas-wyss/engineered-riboregulator-ML/tree/master/data].

Field-specific reporting

Please select the one below that is the best fit for your research. If you are not sure, read the appropriate sections before making your selection.

- [x] Life sciences
- [ ] Behavioural & social sciences
- [ ] Ecological, evolutionary & environmental sciences

For a reference copy of the document with all sections, see nature.com/documents/nr-reporting-summary-flat.pdf

Life sciences study design

All studies must disclose on these points even when the disclosure is negative.

Sample size	For Figures 4G and 4H, sample sizes were chosen as a number that was feasible to test in triplicate while still having enough statistical power for comparisons between groups, with a minimum sample size of n = 4 sequences.
Data exclusions	Data was excluded as per the criteria established in Angenent-Mari et al. 2020. Downsampling was performed as detailed in Supplementary Figure 11 before model training to address abnormally high peaks at some bins in the data.
Replication	For Figures 4G and 4H, all sequences were tested in triplicate. For computational analyses, code has been made available to reproduce the relevant results and figures.
Randomization	Scrambled and shuffled sequences were generated by using built-in randomization functions. All random samples of the toehold experimental data for plotting purposes were also selected via standard randomization functions.
Blinding	Blinding was not relevant to our study.

Reporting for specific materials, systems and methods

We require information from authors about some types of materials, experimental systems and methods used in many studies. Here, indicate whether each material, system or method listed is relevant to your study. If you are not sure if a list item applies to your research, read the appropriate section before selecting a response.

Materials & experimental systems

n/a	Involved in the study	
x		Antibodies
x		Eukaryotic cell lines
x		Palaeontology and archaeology
x		Animals and other organisms
x		Human research participants
x		Clinical data
x		Dual use research of concern

Methods

n/a	Involved in the study	
x		ChIP-seq
x		Flow cytometry
x		MRI-based neuroimaging
