# Supplementary material for: Sequence-to-function deep learning frameworks for engineered riboregulators
Source: Nat Commun. 2020 Oct 7;11:5058. doi: 10.1038/s41467-020-18676-2 (PMC7541510; doi:10.1038/s41467-020-18676-2)
Supplement: Supplementary file 4 — Description of Additional Supplementary Files [file 41467_2020_18676_MOESM4_ESM.pdf]

**Title:** Supplementary Data 1

**Description: Convolutional model performance was optimized via a hyperparameter random grid search.** To find the optimal convolutional model architecture, several hyperparameters were varied and randomly sampled. For all hyperparameter combinations, $R^2$ and Spearman correlation coefficients were evaluated across both ON and OFF values to ensure model predictions are sufficiently consistent with experimental results.
